# TriPerceptNet: a lightweight multi-scale enhanced YOLOv11 model for accurate rice disease detection in complex field environments

**DOI:** 10.3389/fpls.2025.1614929

**Published:** 2025-09-04

**Authors:** Xin Zhang, Linjing Wei, Ruqiang Yang

**Affiliations:** College of Information Science and Technology, Gansu Agricultural University, Lanzhou, Gansu, China

**Keywords:** rice disease detection, YOLOv11, TMLPM, C3K2 MSEIE, ADown, SimAM, lightweight CNN, GradCAM++

## Abstract

This study proposes EDGE-MSE-YOLOv11, a novel lightweight rice disease detection model based on a unified Tri-Module Lightweight Perception Mechanism (TMLPM). This mechanism integrates three core components: multi-scale feature fusion (C3K2 MSEIE), attention-guided feature refinement (SimAM), and efficient spatial downsampling (ADown), which significantly enhance the model’s ability to detect multi-scale and small disease targets under complex field conditions. Unlike isolated architectural enhancements, TMLPM supports collaborative feature interactions, which significantly improves the interpretability and computational efficiency of the model under complex environmental conditions. Experimental results show that, compared with the baseline YOLOv11n model, EDGE-MSE-YOLOv11 improves precision (from 85.6% to 89.2%), recall (from 82.6% to 86.4%), mAP@0.5 (from 90.2% to 92.6%), and mAP@0.5:0.95 (from 63.7% to 70.3%). The model also reduces parameter count by 0.69M and computational cost by 0.3 GFLOPs, while maintaining a high inference speed of 111.6 FPS. These results validate its effectiveness in identifying small, dense lesion areas with high accuracy and efficiency. However, the model still faces challenges in detecting ultra-small or occluded lesions under extremely complex conditions and has yet to be evaluated across multiple domains. Future work will focus on cross-domain generalization and deployment optimization using lightweight techniques such as quantization, pruning, and transformer-based enhancements, aiming to build a robust and scalable disease diagnosis system for intelligent agriculture.

## 1 Introduction

Crop diseases are a major limiting factor that significantly hampers agricultural productivity (Savary et al., 2012). The implementation of precise pest and disease control strategies is essential to simultaneously increase crop yield and overall production capacity (Lucas, 2011). As a staple global food crop, rice production has long been threatened by fungal and bacterial diseases such as rice blast, bacterial blight, and brown spot, which can reduce yields by 20-50%. These diseases pose a significant threat to global food security. Rapid identification and targeted treatment of rice diseases play a crucial role in ensuring stable yields (Mew et al., 2004).

To effectively address the growing challenges posed by crop diseases, improving the accuracy and efficiency of plant disease detection has become a key research focus (Martinelli et al., 2015). Deep learning-based object detection methods, particularly those based on the YOLO series, have shown great promise due to their balance between speed and accuracy (Wang et al., 2025d; Shoaib et al., 2023). In recent years, numerous improvements have been made to YOLO architectures through module enhancements, attention mechanisms, and lightweight strategies.

Several studies improved YOLOv4–YOLOv7 to boost detection performance in complex environments. For example, Fu et al. (2021) introduced CBAM into YOLOv4 to enhance marine target detection, addressing small and overlapping object issues. Mathew and Mahesh (2022) used YOLOv5 with mosaic augmentation for bacterial spot detection in bell peppers. Soeb et al. (2023) adopted YOLOv7 with E-ELAN and mosaic data augmentation to detect tea leaf diseases efficiently.

The release of YOLOv8 triggered a wave of attention-focused enhancements in crop disease detection. Ye et al. (2024) developed YOLOv8-RMDA using RFCBAM and MixSPPF to detect small tea leaf disease targets. Yang et al. (2024) proposed an improved YOLOv8n with C2f_MSEC and BiFPN for rice false smut detection. Similarly, Xue et al. (2023) and Li et al. (2024) enhanced YOLOv5 and YOLOv8 with ACmix, RFB, and GhostNet to improve detection of tea and maize leaf diseases, respectively. Wang et al. (2025c) designed RGC-YOLO with CBAM and GhostConv for multi-scale rice disease detection, achieving 93.2% mAP_50_ with reduced parameters and inference cost.

Recent efforts focus on YOLOv10/11 for real-time agricultural deployment. Guan et al. (2024) enhanced YOLOv10 with BiFPN and GCNet for wheat spike detection, while Huang et al. (2025b) proposed YOLO-YSTs for pest detection on sticky traps using SPD-Conv and Inner-SIoU. YOLOv11-based studies include: Lee et al. (2025) applied hyperparameter optimization to YOLOv11m for tomato disease detection; Zhang et al. (2025) developed YOLO11-Pear for complex orchard pear detection; Sapkota and Karkee (2024a) integrated CBAM into YOLO11x-seg for seasonal tree segmentation; Rao et al. (2025) proposed a lightweight YOLO11 model using Efficient Multi-scale Attention and CMUNeXt for defect detection; Kutyrev et al. (2025) employed YOLO11x for UAV-based apple counting.

In addition to agricultural-specific models, recent literature has explored lightweight and high-performance detection frameworks across various domains, providing valuable inspiration for agricultural visual recognition. For example (Wang et al., 2025a), proposed an embedded cross framework with a dual-path transformer for high-resolution salient object detection, achieving state-of-the-art results on public datasets. However, their method targets generic salient object segmentation and is not specifically optimized for agricultural disease detection or the identification of small, irregular lesions in crops (Zhou et al., 2023). developed an improved YOLACT-based instance segmentation approach for accurate and real-time ice floe identification in polar remote sensing imagery. However, their work focuses on sea ice segmentation rather than the detection of small lesions in agricultural settings (Chen et al., 2025). proposed a dual-pathway instance segmentation network (TP-ISN) for rice row detection, featuring a lightweight backbone and real-time deployment on embedded devices. However, their method mainly addresses structured crop row extraction rather than the detection of small, irregular lesions in crop disease scenarios (Jiang et al., 2025 presented a real-time poultry tracker leveraging the SimAM attention mechanism within YOLOv5 to improve localization of moving animals. Although SimAM enhances key region focus, their approach is tailored to animal tracking and does not specifically optimize for the fine-grained, small lesion detection required in crop disease analysis (Deng et al., 2025). proposed a CBAM-enhanced ResNet framework for 3D circle recognition in point cloud data, achieving precise geometric feature extraction but at the cost of increased computational burden, which limits its real-time and edge deployment applicability.

Although considerable advancements have been made in YOLO-based plant disease detection, most existing models focus on optimizing isolated components while overlooking the synergistic potential of integrated architectural innovations. To address this gap, this study proposes a Tri-Module Lightweight Perception Mechanism (TMLPM)—a unified framework that incorporates multi-scale edge-aware feature fusion (C3K2_MSEIE), attention-guided feature refinement (SimAM), and efficient spatial downsampling (ADown). Rather than relying on single-point enhancements, TMLPM emphasizes coordinated module interaction to enable robust, high-resolution detection of dense, small-scale lesions in real-world agricultural environments. The resulting EDGE-MSE-YOLOv11 model, built upon TMLPM, achieves a superior trade-off between detection accuracy and computational efficiency, and demonstrates real-time performance in practical deployments under complex field conditions.

Building upon this architecture, we further develop EDGE-MSE-YOLOv11, a lightweight and high-performance object detection model specifically designed for rice disease recognition under complex and variable field conditions. Based on YOLOv11n, the proposed model integrates edge-enhanced multi-scale feature extraction, efficient attention mechanisms, and optimized downsampling strategies to improve detection accuracy while maintaining low computational overhead.

The proposed model has been successfully deployed as a client-side application for real-time disease detection in agricultural environments. This implementation enables efficient disease detection with minimal computational overhead, ensuring that users can deploy the model on client devices with acceptable performance. Currently, the system processes image data from agricultural cameras and other sensors for real-time analysis. In the future, as smart agriculture applications continue to evolve, we plan to extend the model to mobile platforms, offering broader accessibility and facilitating on-site disease detection in the field using smartphones for agricultural practitioners. The primary contributions of this work are summarized as follows:

a. This study proposes a novel Tri-Module Lightweight Perception Mechanism (TMLPM), which integrates three complementary strategies—multi-scale edge feature enhancement, attention-guided refinement, and efficient downsampling—into a unified detection framework. This design enables collaborative feature interaction and provides a new direction for constructing high-performance lightweight detectors in agricultural scenarios.
b. A comprehensive and large-scale rice disease dataset has been curated, encompassing six prevalent disease types under diverse field conditions. This dataset supports robust training and evaluation of deep detection models.
c. Extensive ablation studies and comparative experiments were conducted to validate the effectiveness and generalization ability of the proposed model. Results show superior performance over existing lightweight detectors in both accuracy and inference speed.
d. A real-time visualization system based on the proposed model was implemented, demonstrating its practical applicability in field deployment scenarios for precision agriculture.

In summary, this study introduces the concept of Tri-Module Lightweight Perception Mechanism (TMLPM), which synergistically integrates edge-enhanced multi-scale representation (C3K2 MSEIE), spatial-semantic refinement (SimAM), and computationally efficient downsampling (ADown). This mechanism serves as a unified structural foundation for constructing high-performance, low-complexity detection models, marking a significant step forward in the lightweight evolution of vision models for agricultural applications.

## 2 Construction of the recognition model

### 2.1 YOLOv11 overview and algorithm improvements

The YOLO family encompasses a series of deep learning-driven architectures tailored for real-time object detection tasks. These models identify object positions and classes in a single forward pass across an image (Sirisha et al., 2023). The most recent version, YOLOv11n, released by Ultralytics on September 30, 2024, represents the latest advancement in this line of detection models (Jegham et al., 2024), offering improvements in precision, processing speed, and computational efficiency. Significant architectural updates include substituting the conventional C2f module with the C3K2 module in the backbone to enhance feature extraction capabilities, and integrating the C2PSA module to strengthen multi-scale feature learning. Additionally, the revamped spatial pyramid pooling combined with C2PSA increases the diversity of feature representations (Khanam and Hussain, 2024). The network neck employs a PAN-FPN structure to effectively merge low- and high-level features, thereby refining localization performance. The detection head incorporates a decoupled architecture with depthwise separable convolutions (DWConv) to reduce both parameter count and computational overhead. In the YOLOv10n variant, “n” refers to “nano”. YOLOv11n builds on this design by accelerating inference and minimizing model size through streamlined layers and parameters, which enhances deployment feasibility on hardware-limited platforms. In this study, YOLOv11n is adopted as the baseline due to its balance of efficiency and performance. Compared to earlier versions such as YOLOv8n and YOLOv10n, YOLOv11n achieves a 2% gain in inference speed, while reducing resource consumption, making it particularly suitable for real-time detection tasks on constrained devices (Sapkota et al., 2024). Hence, YOLOv11n serves as the foundational architecture for the proposed rice disease identification framework. The overall structure of YOLOv11 comprises four essential components: input, backbone, neck, and head, as illustrated in **Supplementary Figure S1**.

**Figure 1:**
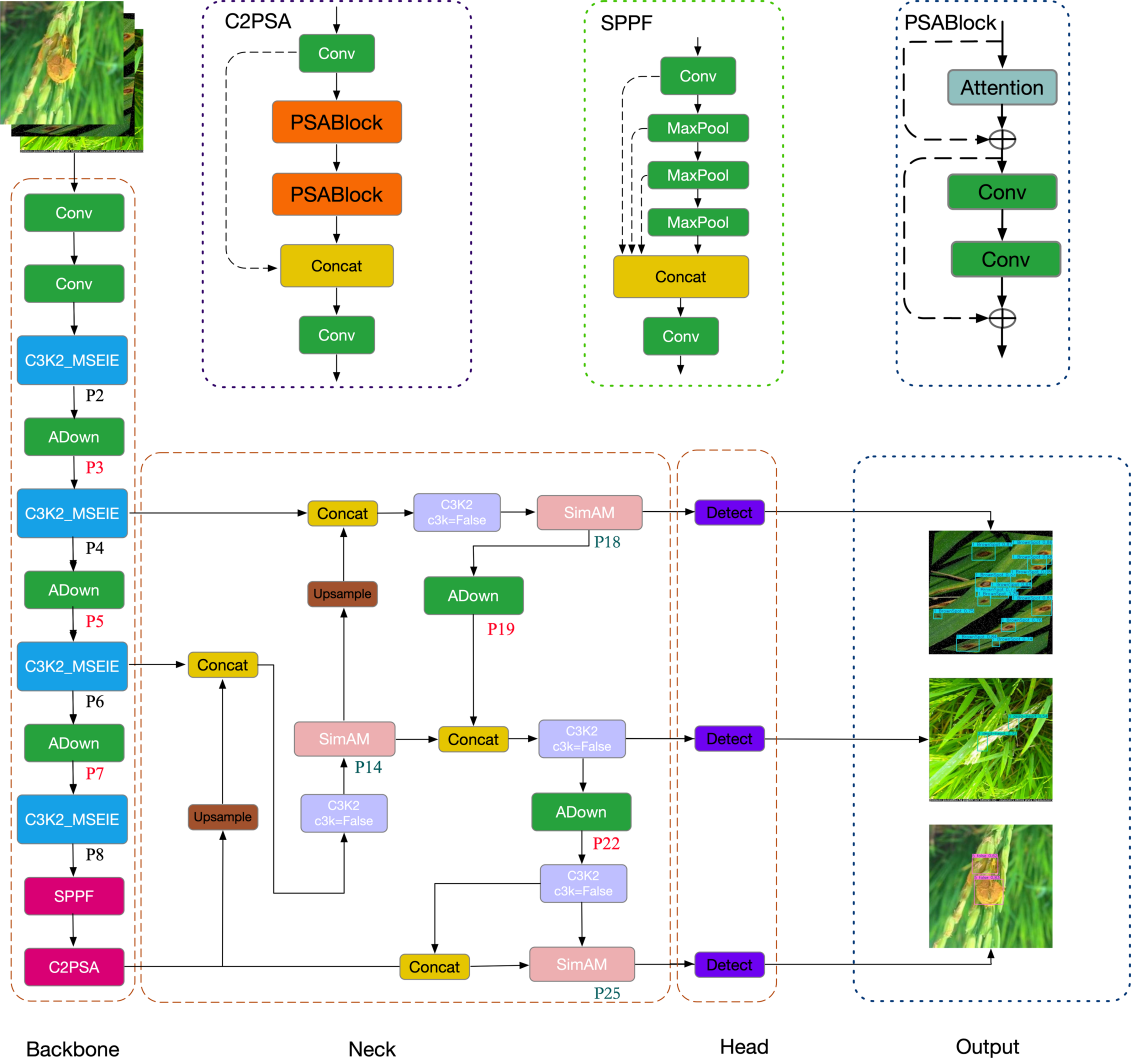
EDGE-MSE-YOLOv11 model structure diagram.

Although YOLOv11 demonstrates strong detection performance, its application in rice disease detection 137 still requires refinement to improve both accuracy and efficiency—especially for small lesion regions, 138 which are typically low in contrast, irregular in shape, and easily lost during downsampling. To address 139 this challenge, this study proposes EDGE-MSE-YOLOv11, a lightweight and enhanced version of 140 YOLOv11n specifically optimized for detecting small objects in rice disease scenarios. The overall 141 architecture, illustrated in **Figure 1**, consists of an improved Backbone, an optimized Neck, and the original 142 Head.

The Backbone network incorporates several targeted enhancements to improve feature extraction and localization of small lesions: First, the standard C3K2 modules in layers 2, 4, 6, and 8 are replaced with the proposed C3K2_MSEIE (MultiScale Edge Information Enhance) module. This structure enhances edge-aware multi-scale feature fusion, which is particularly effective in preserving lesion boundaries and improving the discrimination of small and subtle disease spots. Second, to minimize the loss of fine-grained spatial features during downsampling, traditional convolutional layers at positions 3, 5, 7, 19, and 22 are replaced with the ADown module. This lightweight design preserves critical lesion details and contributes to improved detection accuracy for small diseased regions.

The Neck network is further strengthened by integrating the SimAM attention mechanism in layers 14, 18, and 25. This module enhances the model’s ability to focus on lesion-relevant regions by suppressing background noise and emphasizing discriminative features—especially useful for small lesion areas—without increasing computational overhead.

### 2.2 C3K2 Integrating MSEIE (Multi Scale Edge Information Enhance)

In rice disease detection, significant challenges arise due to large variations in lesion scale, complex and irregular shapes, and cluttered backgrounds. Traditional convolutional neural networks (CNNs), which rely on fixed-grid convolutional kernels, often fail to effectively adapt to such variations in scale and geometry (He et al., 2021). This limitation becomes especially pronounced when detecting small, deformed, or edge-ambiguous lesions, resulting in reduced detection accuracy and poor robustness in real-field scenarios.

**Figure 2:**
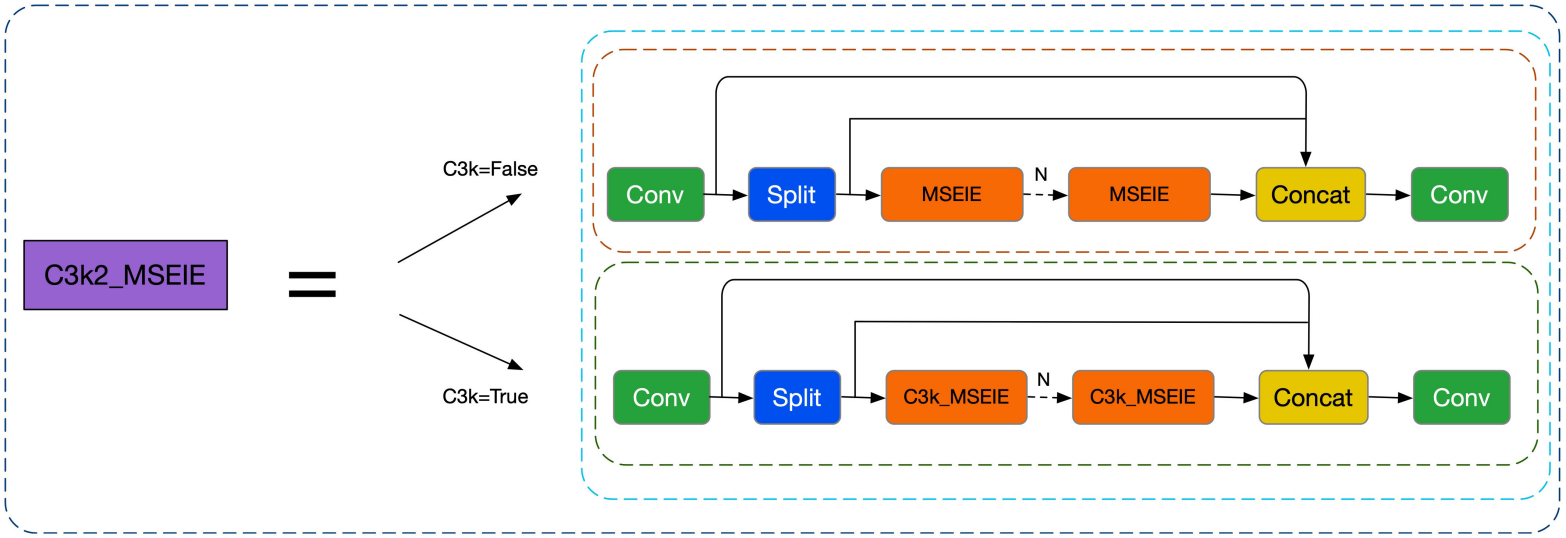
C3K2 MSEIE structure diagram.

**Figure 3:**
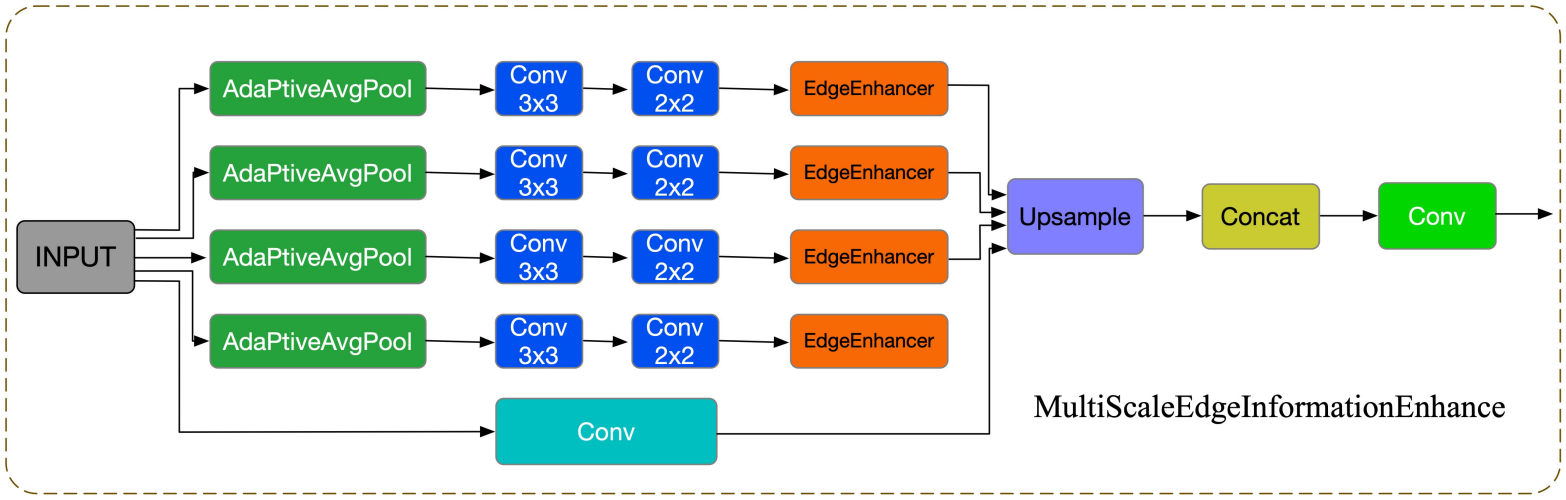
MSEIE structure diagram.

To address these issues, this study introduces a novel module called Multi-Scale Edge Information Enhancement (MSEIE), which is embedded into the C3K2 structure of YOLOv11n (Zhao et al., 2025), forming the improved C3K2_MSEIE module, as illustrated in **Figure 2**. The MSEIE module is designed to enhance the model’s ability to detect small and irregular lesions by combining three core functions: multi-scale feature extraction, edge information enhancement, and efficient feature fusion. As shown in **Figure 3**, it extracts features at multiple receptive fields to better handle scale variations, amplifies critical edge contours to improve boundary localization, and fuses the resulting features to form robust representations.

This targeted design enables the network to retain fine-grained spatial and boundary information while maintaining lightweight efficiency. The integration of MSEIE significantly boosts the model’s performance in detecting rice disease lesions of varying scales and shapes, thereby improving both detection accuracy and overall robustness. The main computational process of the MSEIE module is outlined as follows:

#### 2.2.1 Multi-scale feature extraction

The module first employs Adaptive Average Pooling (AdaptiveAvgPool2d) to achieve multi-scale pooling, extracting local information of different sizes to generate multi-scale feature maps with rich hierarchical structures (Liu et al., 2022). Given an input feature map χ*∈* $R^{C\times H\times W}$ , multiple scale feature maps are obtained through adaptive average pooling, as shown in Equation 1:


(1) $$X_{8}=\text{AdaptiveAvgPool}2\text{d}\left(X\right),S\in \left\{S_{1},S_{2},\ldots ,S_{n}\right\}$$

Where $X_{8}$ represents the pooled feature map at scale S , with the subscript “8” indicating that the spatial 180 resolution of the pooled output is 8 × 8, obtained by applying adaptive average pooling with an output size 181 of 8 × 8.

#### 2.2.2 Edge information enhancement

**Figure 4:**
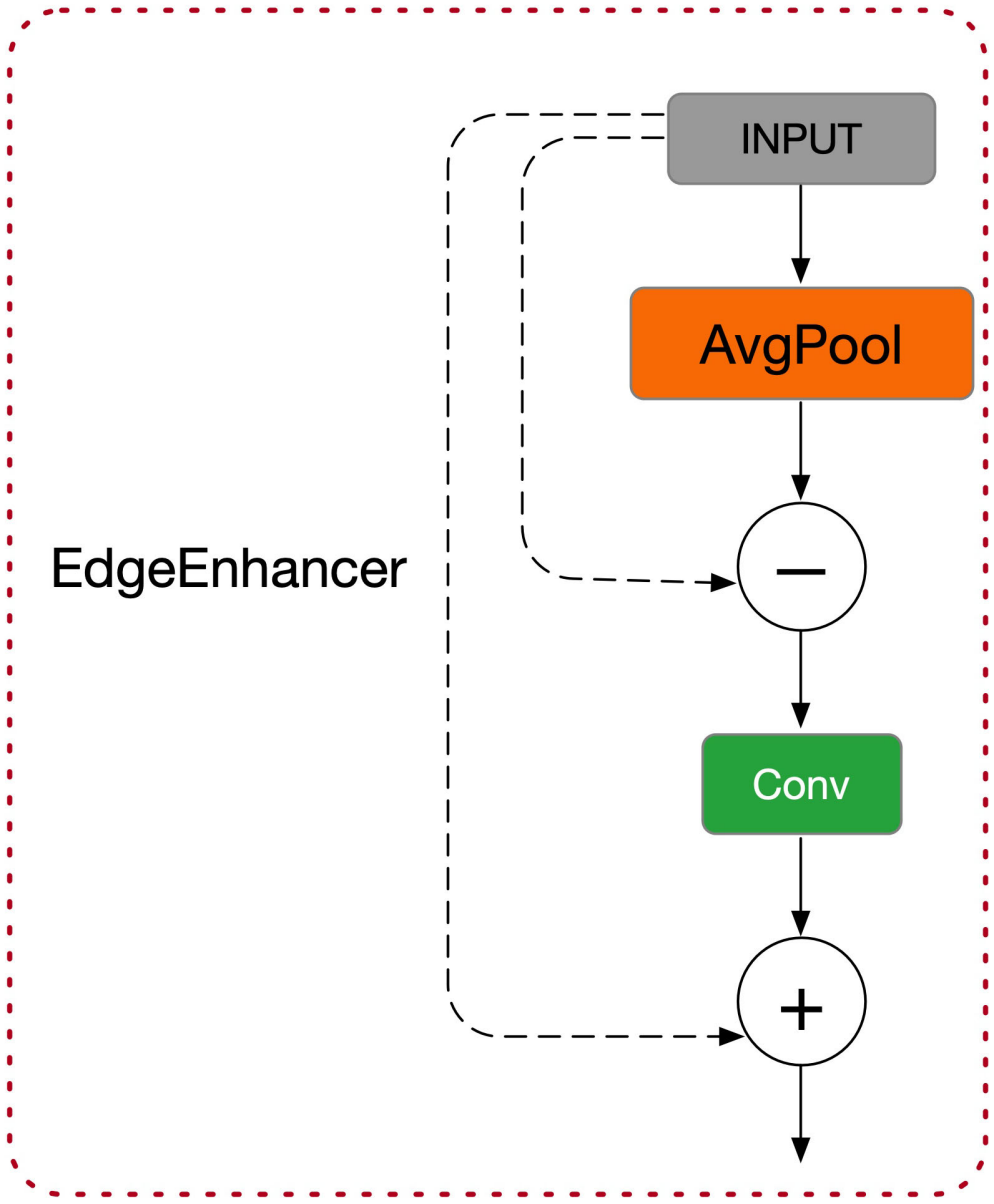
EdgeEnhancer structure diagram.


Building on this, the module introduces EdgeEnhancer (as shown in **Figure 4**) to extract edge information from the image (Suzuki et al., 2003). The core idea is to compute local gradient changes to enhance edge information, using the Laplacian Transform to highlight edge features, as shown in Equation 2:

(2) $$ℰ=\nabla^{2}X$$

The feature map after edge enhancement is denoted as ℰ, and ∇^2^ represents the Laplacian operator. This operation notably increases the network’s sensitivity to edge regions, enhancing the accuracy of object detection tasks.

#### 2.2.3 Feature fusion and output

To fully leverage the feature information across different scales, the MSEIE module adopts a feature fusion strategy, integrating features of various dimensions through channel concatenation and convolution operations, as shown in Equation 3:


(3) $${ℱ}_{\text{out}}=\sigma \left(W_{f}\left[X_{1},X_{2},\ldots ,X_{n},ℰ\right]\right)$$

In this context, [·] indicates the operation of channel-wise concatenation, *W_f_* represents the convolutional kernel applied to the fused features, and *σ*(·) denotes the non-linear activation function, such as ReLU or SiLU. This mechanism facilitates the efficient integration of features across multiple scales, resulting in a unified feature representation derived from the convolutional output. Consequently, the model’s capacity to recognize objects of varying sizes is significantly improved. The C3K2 MSEIE module further enhances detection performance for small-scale objects and targets embedded in complex scenes by leveraging multi-scale pooling, edge-aware enhancement, and advanced feature fusion strategies. These improvements contribute to increased precision and robustness in the context of rice disease recognition.

### 2.3 The SimAm attention mechanism is introduced into the neck network

Detecting densely distributed small objects in cluttered environments remains a key challenge in modern object detection, especially in agricultural scenarios such as rice disease recognition. Variations in object scale, irregular lesion shapes, and complex backgrounds often prevent conventional detectors from focusing on critical features, leading to decreased accuracy and robustness.

**Figure 5:**
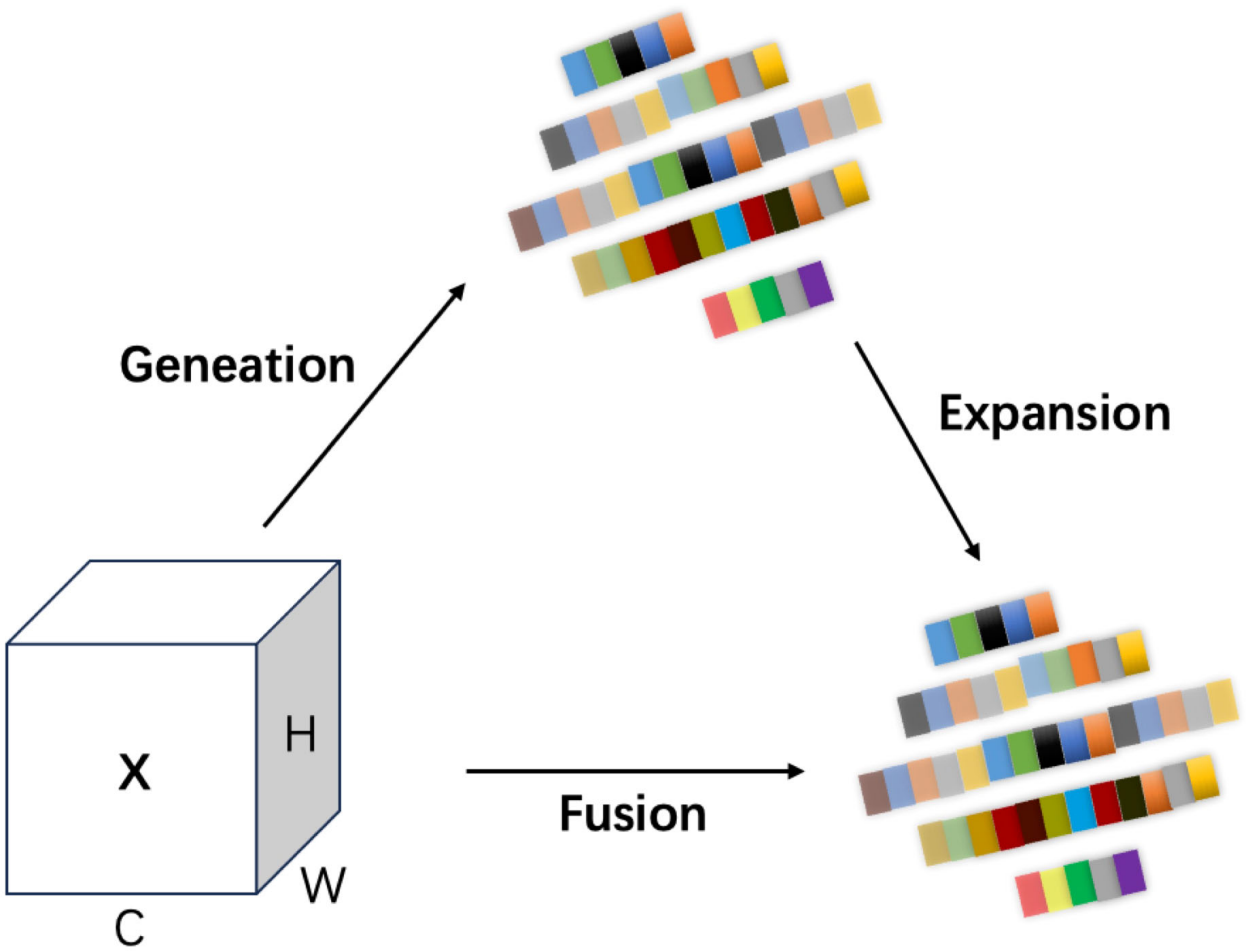
SimAm structure diagram.

To address this limitation, this study incorporates the SimAM (Similarity Attention Module) mechanism into the model architecture, as illustrated in **Figure 5**. SimAM is a lightweight, parameter-free attention mechanism that enhances feature representations by evaluating the self-similarity between spatial elements in the feature map (Suzuki et al., 2003). It operates on the assumption that pixels with similar context should be assigned higher attention, while dissimilar or noisy regions are suppressed. By quantifying the similarity between each pixel and its local neighborhood (Xiang et al., 2024), SimAM highlights salient lesion features and attenuates background interference, which is crucial for detecting small and visually subtle disease spots.

Compared to conventional attention mechanisms such as SE (Squeeze-and-Excitation) and CBAM (Convolutional Block Attention Module), SimAM provides substantial advantages in both efficiency and accuracy (Wu and Dong, 2023; Yan et al., 2024). SE only performs channel-wise weighting and lacks spatial adaptability, making it ineffective for localizing small-scale lesions in complex scenes. Furthermore, its fully connected layers increase computational overhead. CBAM introduces both spatial and channel attention but often suffers from high latency, with its integration into architectures like ResNet50 resulting in up to a 15% slowdown in inference—unsuitable for real-time or edge deployments.

Given the hardware constraints and the need for precise lesion localization in rice disease detection, SimAM is selected as the primary attention mechanism in this study. It offers enhanced discriminative focus on disease regions while preserving model compactness, reducing false detections and improving overall robustness under complex field conditions.

To implement SimAM, the mechanism evaluates attention by computing pixel-level similarity scores. For an input feature map $X\in {\mathbb{R}}^{B\times C\times H\times W}$ , where B is the batch size, C the number of channels, and H , W the height and width respectively, SimAM first normalizes each feature vector, then computes the similarity between a given pixel and all others in the same feature map. The similarity score is used to reweight each spatial location, producing an updated feature representation that emphasizes contextually important regions. The complete computation process of SimAM is defined as follows:

First, the mean $\mu \in {ℛ}^{ℬ\times C\times 1\times 1}$ and variance $v\alpha \gamma \in {ℛ}^{ℬ\times C\times 1\times 1}$ of the feature map X are computed along the height and width dimensions. Then, the feature map X is standardized to obtain the normalized feature map $\tilde{x}\in {ℛ}^{ℬ\times C\times 1\times 1}$ , as shown in Equation 4:


(4) $$\tilde{x}=\frac{x-\mu }{\sqrt{v\alpha \gamma +\epsilon }}$$

In which *ϵ* is a very small constant to prevent the denominator from being zero.

The similarity $Y_{i,j}$ between each pixel $X_{i,j}\in \tilde{x}$ and all other pixels is computed and normalized. For each pixel $X_{i,j}$ , the similarity values with other pixels are computed as shown in Equation 5:


(5) $$Y_{i,j}=\frac{X_{i,j}^{2}}{4\left(\frac{1}{n-1}\sum_{k\neq i,j}x_{k}^{2}+\epsilon \right)}+0.5$$

The number of pixels in the feature map is denoted as *n* = *HW*. The similarity *Y_i,j_* is computed by normalizing the squared difference between *X_i,j_* and all other pixels, followed by adding a bias term of 0.5, mapping the similarity value to the range [0,1]. The original feature map *X* is then multiplied element-wise by the similarity *Y*, yielding the weighted feature map Z *∈* $R^{B\times C\times W\times H}$ , where B is the batch size, and the feature map value is computed as: *Z_i,j_* = *X_i,j_Y_i,j_*. Subsequently, the weighted feature map Z is normalized using the Sigmoid activation function to obtain the final output feature map. SimAM is a parameter-free, three-dimensional attention mechanism inspired by neuroscientific principles, designed for image classification tasks. Its core concept leverages similarity information to adaptively modulate the attention weights of each channel, thereby enhancing image classification performance.

### 2.4 Introduction of the lightweight ADown module

Balancing detection accuracy and model compactness remains a key challenge in deploying deep learning models, especially in resource-constrained scenarios. While conventional backbone networks such as ResNet and Darknet achieve high detection performance, their large parameter counts and deep convolutional stacks lead to increased inference latency, making them less suitable for lightweight applications such as rice disease detection on edge devices (Tank et al., 2025).

In this context, we propose a novel ADown convolution structure, designed to mitigate feature degradation during downsampling and enhance sensitivity to small lesions—one of the primary challenges in rice disease identification (Fang et al., 2024). Unlike traditional downsampling methods that rely solely on strided convolution or max pooling, ADown integrates both average and max pooling to preserve global context while retaining salient details. Given an input feature map X ADown computes, as shown in Equation 6:


(6) $$X_{\text{avg}}=\text{AvgPool}2\text{d}\left(X\right),\;X_{\text{max}}=\text{MaxPool}2\text{d}\left(X\right)$$

Each branch is processed through a convolutional layer as shown in Equation 7:


(7) $$Y_{1}=\text{Conv}\left(X_{\text{avg}}\right),\;Y_{2}=\text{Conv}\left(X_{\text{max}}\right)$$

The final output is produced by concatenating both branches along the channel axis as shown in Equation 8:


(8) $$Y=\text{Concat}\left(Y_{1},Y_{2}\right)$$

This design allows ADown to retain hierarchical and complementary features while performing resolution reduction (Song et al., 2024). In our implementation, ADown replaces traditional downsampling layers in YOLOv11n’s backbone and neck, which previously relied on stride-2 convolutions and pooling for 8 × , 16 × , and 32 × spatial reductions (Zhai et al., 2023). Although effective for general-scale targets, such methods often discard fine-grained spatial cues that are critical for identifying early-stage or small-scale lesions in rice leaves.

**Figure 6:**
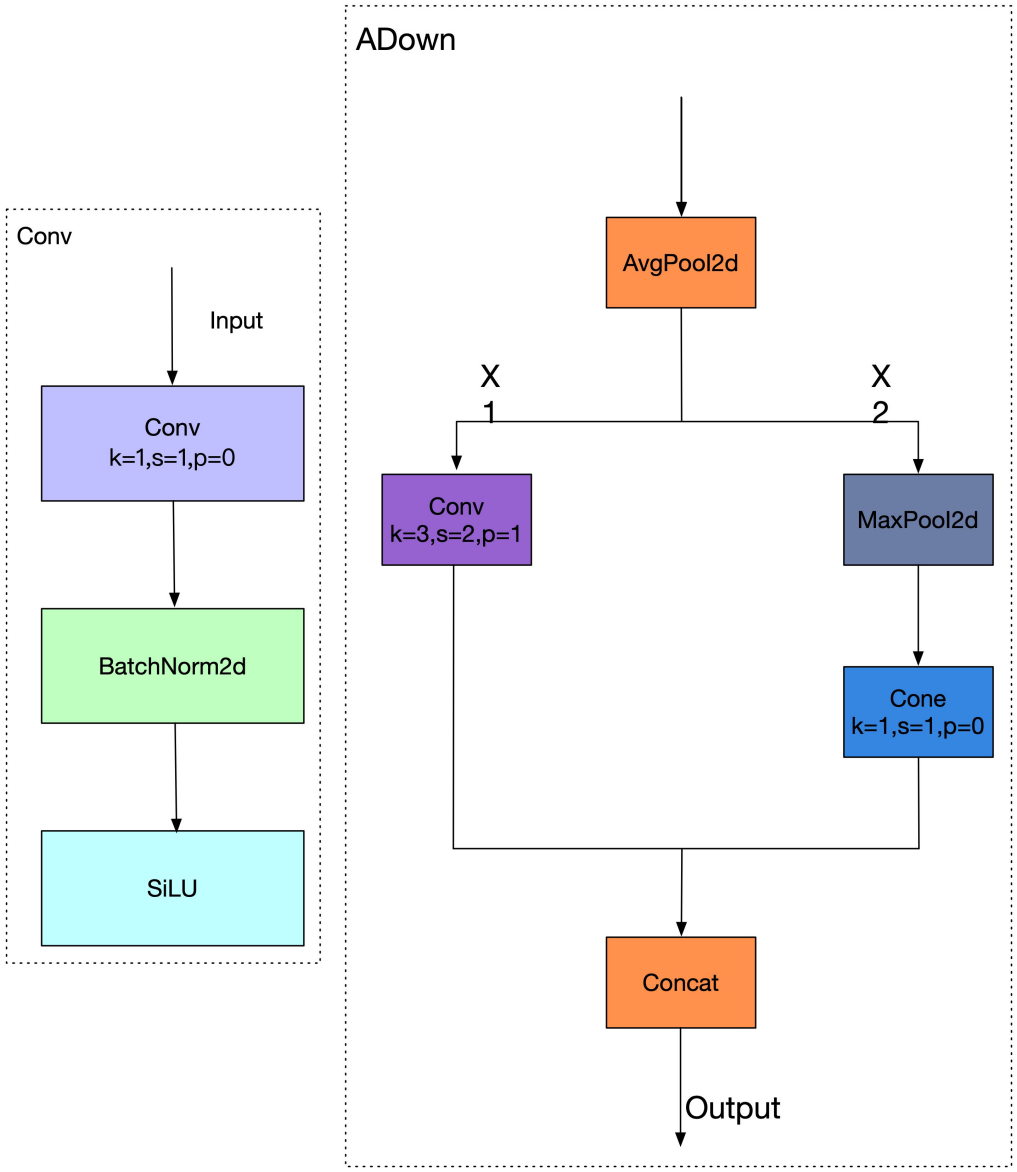
Diagram of conv downsampling and ADown downsampling structures.


To further illustrate the architecture, **Figure 6** compares the traditional convolution-based downsampling workflow (left) with the ADown-enhanced approach (right). In the ADown block, the input feature map is split along the channel axis into $X_{1}$ and $X_{2}$ . The $X_{1}$ branch is convolved directly, while $X_{2}$ undergoes max pooling followed by convolution as shown in Equation 9:

(9) $$Y=\text{Concat}\left(\text{Conv}\left(X_{1}\right),\text{Conv}\left(\text{MaxPool}2\text{d}\left(X_{2}\right)\right)\right)$$

This asymmetric pooling-convolution strategy improves representation capability across scales while 273 maintaining computational efficiency. By replacing conventional downsampling blocks with ADown, our 274 modified YOLOv11n architecture achieves a better trade-off between model size and accuracy. It improves 275 detection precision for small lesions and enhances robustness in cluttered field environments, thereby 276 offering a more lightweight and task-adapted solution for rice disease detection.

## 3 Experimental data and evaluation metrics

### 3.1 Experimental data

**Figure 7:**
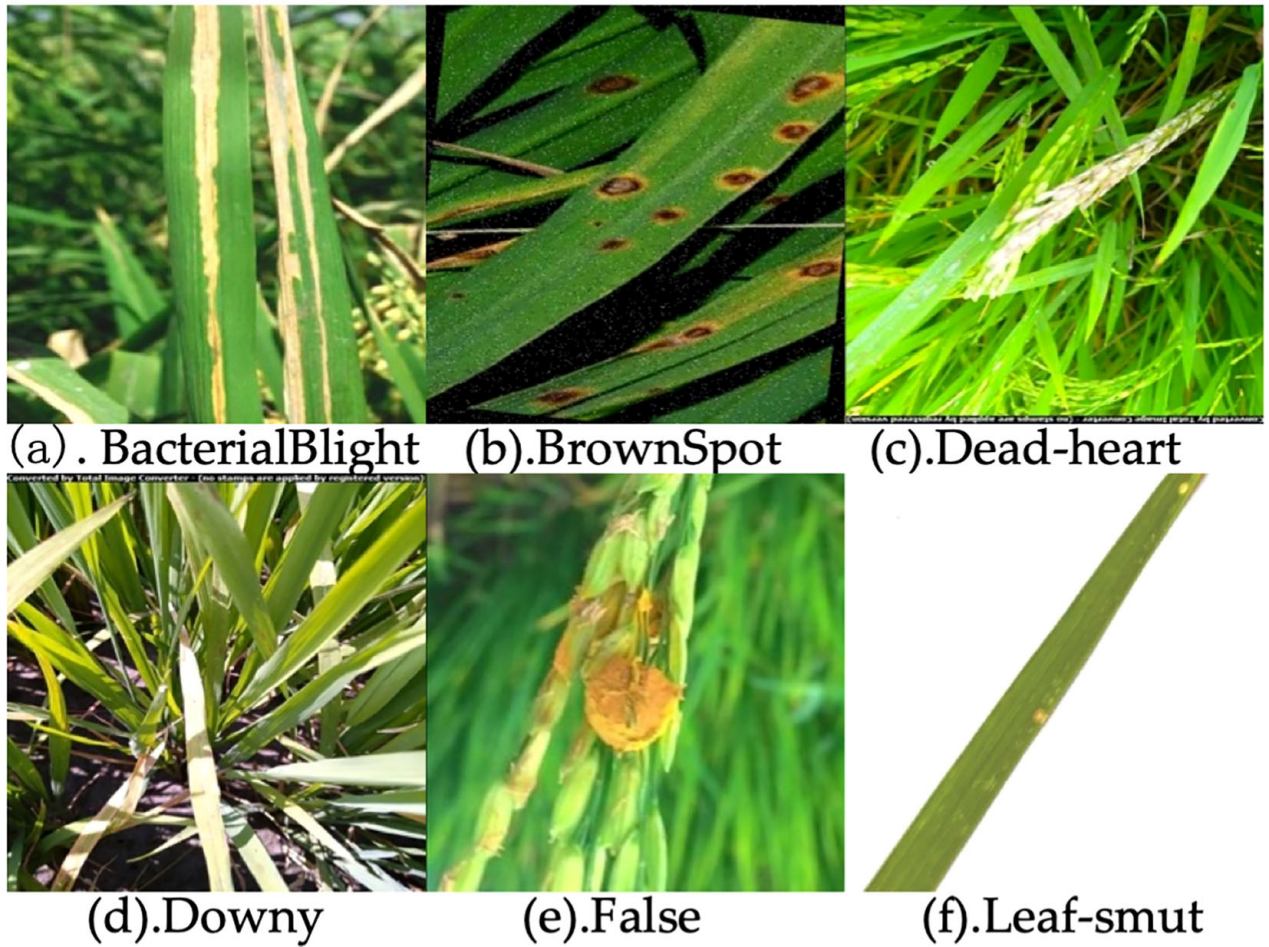
Images of six rice diseases: **(a)** Bacterial Blight, **(b)** Brown Spot, **(c)** Dead-heart, **(d)** Downy, **(e)** False, **(f)** Leaf-smut.

In this study, the dataset was independently collected and encompasses six commonly observed rice diseases, including Bacterial Blight, Brown Spot, Dead-heart, Downy, False, and Leaf-smut (**Figure 7**). To enhance the model’s robustness and adaptability across varying conditions, a series of multi-dimensional geometric transformations and color perturbation techniques were employed during the data preprocessing phase. Specifically, spatial domain augmentation was achieved by applying affine transformation matrices for image displacement, mirror inversion, scaling, and rotation. In addition, color domain augmentation was performed by introducing random perturbations within the HSV color model to dynamically adjust hue, saturation, and brightness, thereby enhancing spectral diversity. As a result, the dataset was expanded to 11,617 images. This composite data augmentation strategy, which simulates visual variations in real-world scenarios, significantly enhanced the model’s feature extraction capability and robustness against interference from unseen samples.

### 3.2 Dataset construction

A total of 11,617 images of rice leaves were collected from experimental paddy fields located in Jiangsu Province, China, during the 2022–2023 growing seasons. Images were captured using a Canon EOS 90D high-definition DSLR camera under various natural lighting conditions to reflect realistic field environments. The dataset covers six common rice diseases: rice blast, bacterial leaf streak, brown spot, sheath blight, leaf blast, and false smut. After collection, the data were carefully filtered to remove duplicates and blurred samples to ensure high-quality input.

Subsequently, the curated dataset was split into training, validation, and testing subsets following a 7:2:1 ratio, as detailed in **Table 1**. Specifically, the training set contains 8,130 images, the validation set 2,324 images, and the testing set 1,163 images. This partition ensures a balanced distribution for model training, validation, and generalization.

**Table 1:** Rice disease dataset.

Disease categories	Training set/Images	Validation set/Image	Test set/Images	Total/Images
Bacterial Blight	1384	334	165	1883
Brown Spot	1208	357	233	1798
Leaf-smut	1221	460	142	1823
Dead-heart	1365	405	195	1965
Downy	1422	322	183	1927
False	1530	446	245	2221

All images were manually annotated by agricultural experts using the LabelImg annotation tool to accurately delineate disease regions. Corresponding YOLO-format ‘.txt’ annotation files were automatically generated for subsequent experiments. The annotation procedure is illustrated in **Supplementary Figure S2**, demonstrating the consistency and standardization of the labeling process. The comprehensive and standardized construction of this dataset provides a solid foundation for robust model training and performance evaluation.

### 3.3 Experimental environment

Experiments were conducted on a cloud server running Ubuntu 20.04, equipped with an NVIDIA RTX 4090 GPU (24 GB) and an Intel^®^ Xeon^®^ Platinum 8358P CPU operating at 2.60 GHz. The software environment comprised CUDA 11.8, PyTorch 2.0.0, and Python 3.8.10.

The model was trained using stochastic gradient descent (SGD) optimizer with an initial learning rate of 0.001, weight decay of 0.0005, and a batch size of 16 over 300 epochs. A cosine annealing learning rate scheduler with warm restarts every 30 epochs was employed to facilitate convergence. Data augmentation techniques, including random horizontal flipping, random cropping, and color jittering, were applied to improve model robustness and generalization. Other hyperparameters were maintained at default settings from the original YOLOv11n implementation to ensure stable and efficient training.

### 3.4 Evaluation metrics

This experiment evaluates the performance of the improved network model from multiple perspectives, including detection accuracy, localization precision, and model complexity. Key metrics such as Precision (P), Recall (R), Mean Average Precision at IoU 0.5 (mAP_50_), Mean Average Precision across IoU thresholds from 0.5 to 0.95 (mAP_95_), parameter count, and computational complexity are used. These metrics provide a comprehensive and objective evaluation of the model’s overall performance.

Precision evaluates the proportion of correctly predicted positive instances among all instances predicted as positive by the model, reflecting its reliability in identifying positive samples, as shown in Equation 10:


(10) $$\text{Precision}=\frac{TP}{TP+FP}$$

Here, TP denotes the number of true positive cases, while FP represents the number of false positive cases.

Recall is a metric that evaluates a model’s ability to detect actual targets, reflecting the proportion of true targets correctly identified, as shown in Equation 11:


(11) $$\text{Recall}=\frac{TP}{TP+FN}$$

Here, FN represents the number of false negatives.

The mean Average Precision (mAP_50_) is the mean of the Average Precision (AP) when the Intersection over Union (IoU) threshold is set to 0.5, as shown in Equation 12:


(12) $$mAP_{50}=\frac{1}{N}\sum_{I}^{N}\int_{0}^{1}P\left(R\right)\;dR$$

The mean Average Precision (mAP_50−95_) is computed as the average of the Average Precision (AP) across IoU thresholds from 0.5 to 0.95, as shown in Equation 13:


(13) $$mAP_{50-95}=\frac{1}{N}\sum_{i=1}^{N}AP_{i}\left(0.95\right)$$

Among them, *AP_i_* represents the Average Precision (AP) for the i-th category, and n denotes the total number of categories.

The number of parameters reflects the storage requirements of the model, specifically the number of weights that need to be trained and stored. A smaller number of parameters leads to a lighter model. Computational cost refers to the computational overhead required by the model during fast inference. Both metrics are crucial for evaluating model usability and are relevant for edge computing devices.

## 4 Experimental results and analysis

### 4.1 Improved module combination effect comparison

To systematically analyze the relationship between model performance and component or design selection, and to validate the effectiveness of the improved modules, this paper uses the original YOLOv11n model as the baseline and sequentially integrates the C3K2 MSEIE module, SimAM module, and ADown module to form the improved model. The results of the ablation experiments are shown in **Table 2**.

**Table 2:** Improved module combination effect comparison.

Detection model	C3K2 MSEIE	SimAM	ADown	P/%	R/%	mAP50/%	mAP95/%	Model size/M	Param/M	Inference speed/FPS	FLOPs/G
YOLOv11n				85.6	82.6	90.2	63.7	4.8	8.55	174.7	5.7
YOLOv11n-A	✓			86.6	85.2	91.8	66.9	5.6	9.69	111.2	6.4
YOLOv11n-B	✓	✓		88	86.3	92.1	68.4	5.6	9.69	111.6	6.4
EDGE-MSE-YOLOv11	✓	✓	✓	89.2	86.4	92.6	70.3	4.4	7.86	102.8	5.4

As shown in **Table 2**, the ablation results clearly demonstrate the effectiveness of each proposed module. Compared to the baseline YOLOv11n model, YOLOv11n-A—constructed by replacing the original C3K2 module with the enhanced C3K2_MSEIE module—achieves notable improvements: precision ( P ) increases from 85.6% to 86.6% (+1.0%), recall ( R ) from 82.6% to 85.2% (+2.6%), mAP_50_ from 90.2% to 91.8% (+1.6%), and mAP_95_ from 63.7% to 66.9% (+3.2%). These gains highlight the effectiveness of multi-scale edge enhancement in improving localization and recognition of fine-grained features.

Building upon this, YOLOv11n-B introduces the SimAM attention mechanism on top of the C3K2_MSEIE-enhanced architecture. This further improves detection metrics, achieving a precision of 88.0%, recall of 86.3%, mAP_50_ of 92.1%, and mAP_95_ of 68.4%. The performance gains confirm that SimAM effectively emphasizes informative regions and suppresses background noise, especially benefiting the detection of small lesion areas.

Finally, the proposed EDGE-MSE-YOLOv11 model, which integrates the C3K2_MSEIE, SimAM, and the lightweight ADown module, achieves the best results across all evaluation metrics: 89.2% precision, 86.4% recall, 92.6% mAP_50_, and 70.3% mAP_95_. This represents an overall improvement of 3.6%, 3.8%, 2.4%, and 6.6%, respectively, over the baseline. These results demonstrate that the joint optimization of feature extraction, attention refinement, and downsampling enhances both accuracy and efficiency, making the model well-suited for rice disease detection in resource-limited environments.

**Figure 8:**
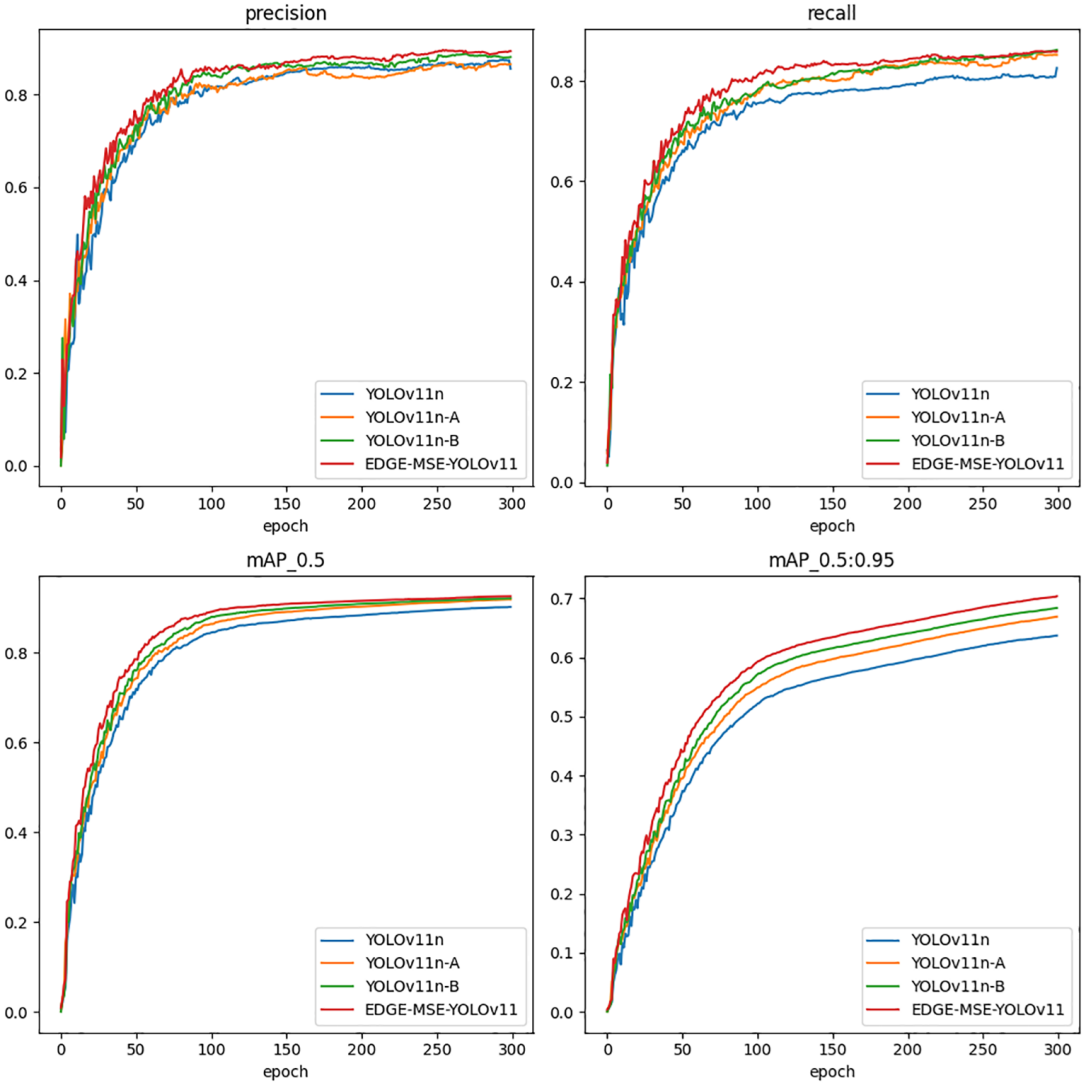
Performance comparison of different models.

**Figure 8** illustrates the performance trends of different models throughout the training process using four standard object detection metrics: precision, recall, mAP@0.5, and mAP@0.5:0.95. Precision measures the ratio of true positives to all predicted positives, reflecting the model’s ability to reduce false detections. Recall indicates the model’s effectiveness in identifying all actual targets. mAP@0.5 evaluates detection accuracy with an Intersection over Union (IoU) threshold of 0.5, while mAP@0.5:0.95 averages performance across IoU thresholds from 0.5 to 0.95, offering a more comprehensive assessment of localization accuracy.

All models exhibit significant initial improvements, rapidly stabilizing after approximately 100 epochs. The EDGE-MSE-YOLOv11 consistently demonstrates superior performance, particularly evident in all four metrics, achieving the highest and most stable values earlier than other models. YOLOv11n-A and YOLOv11n-B also show clear advantages over the baseline YOLOv11n, validating the efficacy of the introduced modules. This significant improvement is attributed to the optimization of the C3K2 module in the backbone network, particularly the introduction of the MSEIE module, which significantly enhances model accuracy. Moreover, replacing the standard downsampling module in the neck network with the lighter ADown module reduces the model’s size and parameters by 0.4% and 0.69%, respectively, while reducing computational cost by 0.3%. Additionally, the inclusion of the SimAM attention module further boosts overall performance. The multi-module fusion EDGE-MSE-YOLOv11 improves detection performance while preserving the model’s lightweight advantage, especially noticeable in the mAP_95_ metric.

**Figure 9:**
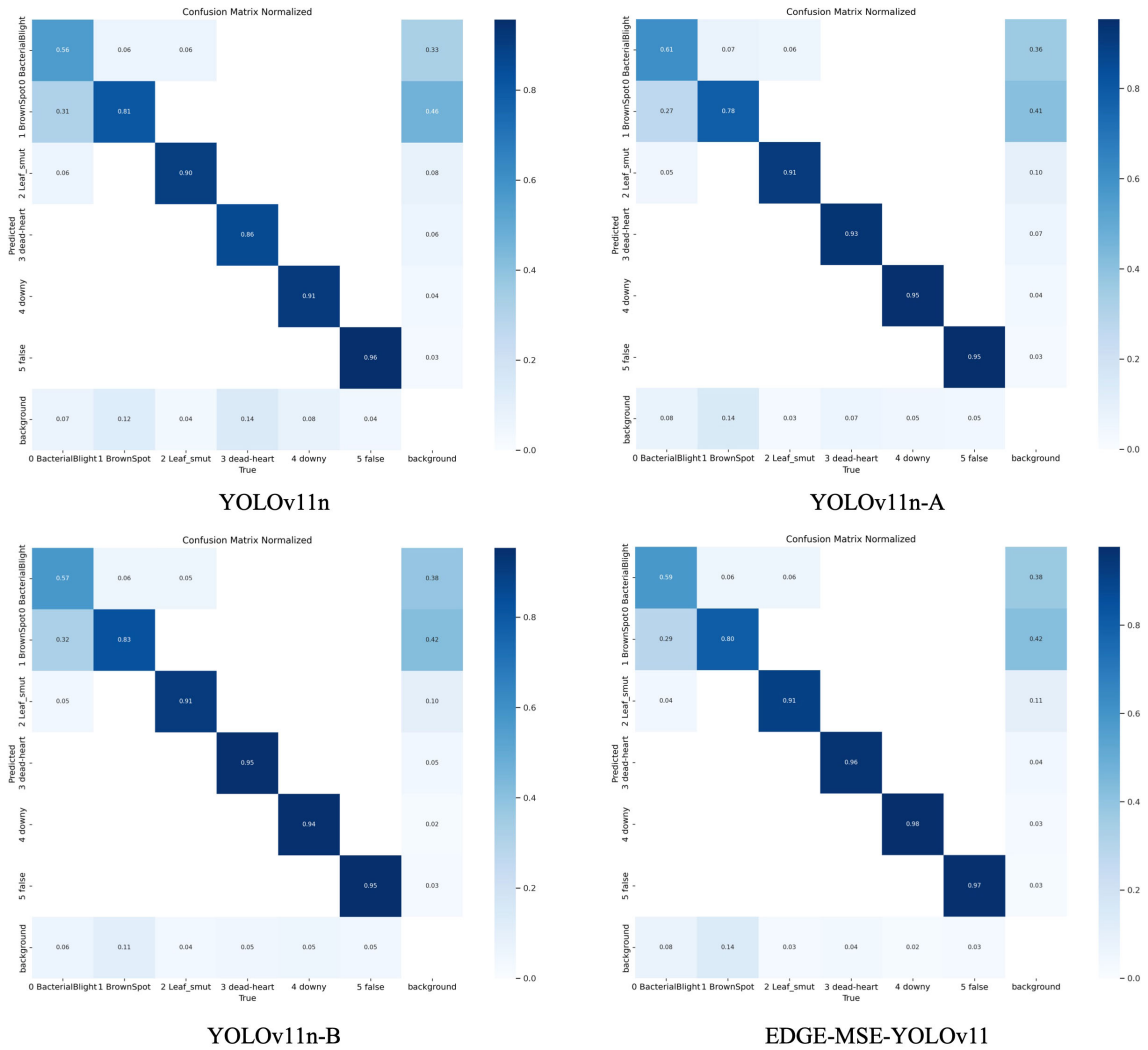
Comparison of confusion matrices before and after model improvement.

**Figure 9** illustrates the confusion matrix for the detection results of rice diseases using the YOLOv11n model series, which includes YOLOv11n, YOLOv11n-A, YOLOv11n-B, and EDGE-MSE-YOLOv11. Comparison of these matrices reveals that they are normalized, with the horizontal and vertical axes representing various disease categories and backgrounds, respectively. Larger diagonal values and darker colors correspond to higher detection accuracy for each category, while off-diagonal elements indicate the proportion of misclassified samples from other categories.

By examining the changes in the confusion matrix, it is evident that as the model progresses from YOLOv11n to YOLOv11n-A, then to YOLOv11n-B, and finally to EDGE-MSE-YOLOv11, the diagonal element colors become progressively darker. This indicates a significant improvement in the model’s recognition accuracy for various diseases. At the same time, the color of the non-diagonal elements lightens, demonstrating the model’s growing ability to differentiate between disease categories and a notable reduction in misclassification rates. For example, earlier versions of the model may have struggled to distinguish between similar diseases, particularly those with similar morphological features. However, these misclassifications were substantially reduced in the later versions. While the YOLOv11n model performs adequately within mainstream lightweight detection frameworks, it still faces challenges in differentiating between similar disease categories and suffers from decreased accuracy under complex background interference. By incorporating enhanced modules like the C3K2 MSEIE, the SimAM attention mechanism, and the ADown downsampling module, the YOLOv11n-A and YOLOv11n-B models show considerable improvement in distinguishing similar diseases. Notably, the EDGE-MSE-YOLOv11 model, which integrates these various enhancements, achieves near-optimal performance. The diagonal elements in its confusion matrix approach a value of 1, while the non-diagonal elements are significantly reduced, demonstrating superior accuracy and robustness. Based on these experimental findings, compared to the baseline model, the proposed EDGE-MSE-YOLOv11 not only improves recognition accuracy for key disease types but also greatly reduces confusion between different diseases, offering a more accurate and reliable solution for rice disease detection in complex environments.

**Figure 10:**
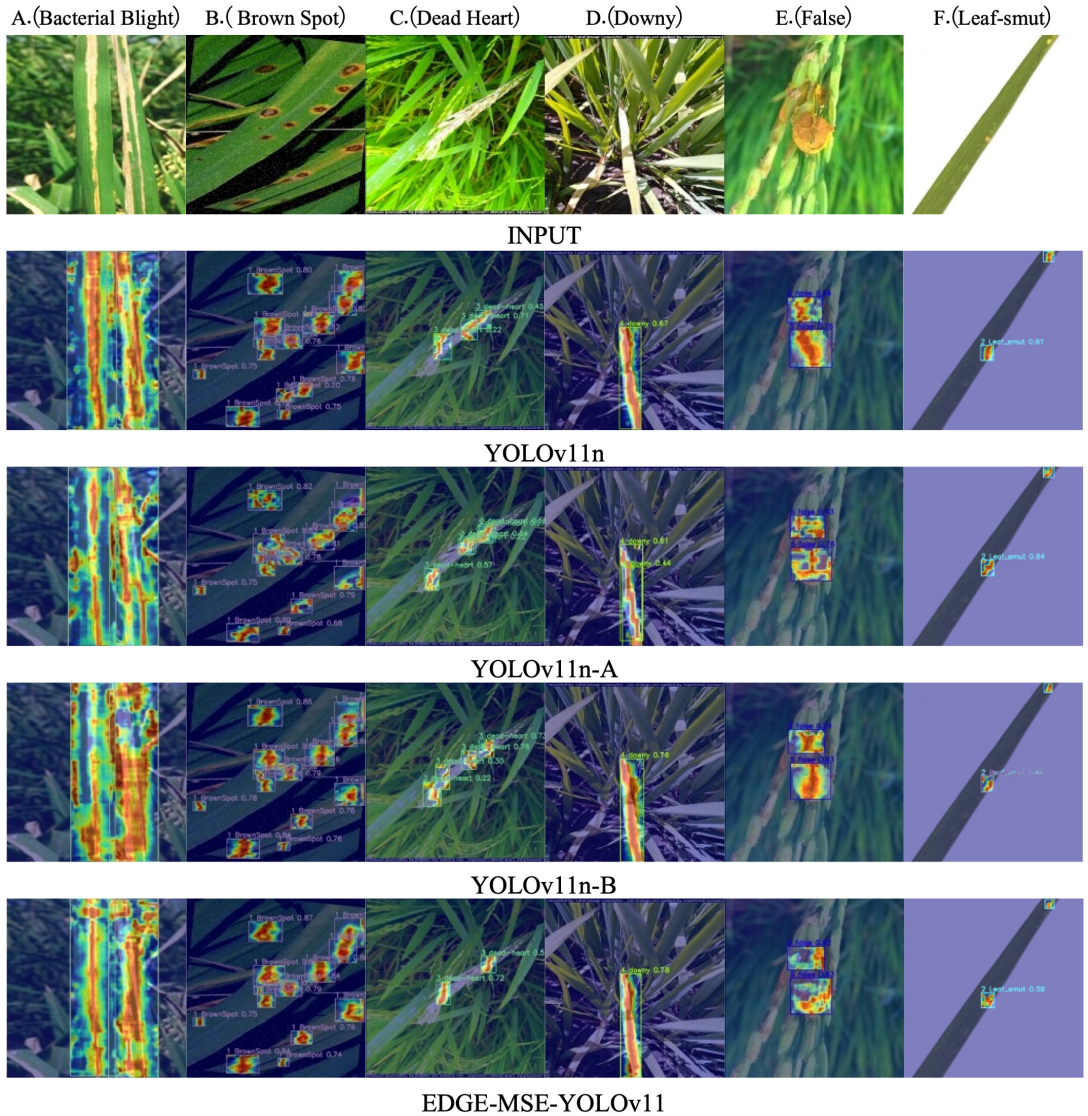
Detection heatmap of the model before and after improvement on the rice disease dataset: **(A)** Bacterial Blight, **(B)** Brown Spot, **(C)** Dead Heart, **(D)** Downy, **(E)** False, **(F)** Leaf-smut.

The Grad-CAM++ technique was employed to visualize the feature activation regions of the models during disease detection (Chattopadhay et al., 2018). The heatmaps shown in **Figure 10** highlight the spatial distribution of model attention within diseased areas of rice leaves. For the original YOLOv11n model, the activation regions appear scattered and disorganized, with a substantial amount of background interference. This dispersion suggests that the model fails to consistently attend to the actual lesion areas, which may result in frequent missed detections. With the introduction of the C3K2 MSEIE module, the YOLOv11n-A model demonstrates noticeable improvements in localizing and emphasizing critical features, particularly in the detection of diseases such as B (Brown Spot), C (Dead Heart), D (Downy), E (False), and F (Leaf-smut). The activation heatmaps become more concentrated around the lesion regions, although challenges remain for class A (Bacterial Blight), where some background textures still exhibit attention. After integrating the SimAM attention mechanism, the YOLOv11n-B model exhibits a substantial reduction in background activation and a sharper focus on diseased regions. This enhanced focus contributes to stronger robustness in complex backgrounds and improved localization of small lesions. Compared to YOLOv11n-A, the TMLPM-enhanced YOLOv11n shows more focused attention on the primary disease locations, enabling faster and more precise identification of target areas. Furthermore, the final EDGEMSE-YOLOv11 model, which incorporates the ADown downsampling module, demonstrates the best trade-off between feature resolution and detection accuracy. The corresponding Grad-CAM++ heatmaps show tightly clustered attention within lesion areas and minimal activation in irrelevant regions. This indicates superior performance in isolating critical lesion features, suppressing background noise, and recognizing small, complex disease patterns. Overall, compared to the baseline YOLOv11n, the TMLPM-driven EDGE-MSE-YOLOv11 offers substantial improvements in lesion localization, feature extraction, and detection robustness. These visualizations further validate the effectiveness of the TMLPM framework in enhancing interpretability and precision in plant disease detection.

### 4.2 Comparison of different attention mechanisms

This study selects YOLOv11n as the baseline model and integrates three attention mechanisms—SE, CBAM, and SimAM—to construct three variant models: YOLO+SE, YOLO+CBAM, and YOLO+SimAM. All models were trained and tested on the same dataset to ensure experimental fairness (As shown in **Table 3**). The results demonstrate that YOLO+SimAM outperforms the other two models in terms of precision, recall, and mean average precision (mAP). Specifically, YOLO+SimAM achieved a precision of 88%, a recall of 86.3%, as well as mAPmAP_50_ and mAPmAP_95_ of 92.1% and 68.4%, respectively, while maintaining a high frame rate of 111.6 FPS and a low computational complexity of 6.4 GFLOPs. In comparison, YOLO+SE attained precision, recall, mAPmAP_50_, and mAP_95_ of 85%, 83.0%, 89.5%, and 65.0%, respectively; YOLO+CBAM achieved 86%, 84.0%, 90.0%, and 66.0% for these metrics. These results clearly indicate that the SimAM attention mechanism has a significant advantage in enhancing the performance of rice disease detection.

**Table 3:** Comparison of different attention mechanisms.

Model	P/%	R/%	mAP50/%	mAP95/%	Model size/M	Param/M	Inference speed/FPS	FLOPs/G
YOLOv11n+SimAM	88.0	86.3	92.1	68.4	5.6	9.69	111.6	6.4
YOLOv11n+SE	85.0	83.0	89.5	65.0	5.9	10.10	108.0	6.8
YOLOv11n+CBAM	86.0	84.0	90.0	66.0	5.8	10.00	109.0	6.7

### 4.3 Comparative experiments on improved models

To comprehensively evaluate the model’s performance, this study conducted a systematic comparative experiment on several mainstream object detection models, including RT-DETR (Zhao et al., 2024), YOLOv5n (Jocher et al., 2021), YOLOv6n (Bist et al., 2023), YOLOv8n (Liu et al., 2023), YOLOv11n (Alkhammash, 2025), and EDGE-MSE-YOLOv11, under unified experimental conditions (Sapkota and Karkee, 2024b). RT-DETR (Wang et al., 2024) is a recent end-to-end object detection framework based on Transformer architecture, offering competitive accuracy and real-time inference capability, which makes it suitable for lightweight agricultural vision tasks. The comparison results are shown in **Table 4**.

**Table 4:** Comparative experiments on improved models.

Model	mAP50/%	mAP95/%	Param/M	FLOPs/G
Rtdetr	83.6	61.6	111.68	105.2
YOLOv5n	88.3	60.1	10.3	7.9
YOLOv6n	86.4	59.6	15.87	11.6
YOLOv8n	90	62.1	8.9	6.2
YOLOv11n	90.2	63.7	8.555	5.7
EDGE-MSE-YOLOv11	92.6	70.3	7.86	5.4

As shown in **Table 4**, compared to standard YOLO series models and the recent Rt-detr model, the EDGE-MSE-YOLOv11 model introduced in this study achieves notably higher detection accuracy while significantly reducing parameter count and computational complexity (Wang et al., 2025b). Specifically, EDGE-MSE-YOLOv11 achieves an mAP_50_ of 92.6% and mAP_95_ of 70.3%, surpassing YOLOv5n by 4.3% and 10.2%, YOLOv6n by 6.2% and 10.7%, YOLOv8n by 2.6% and 8.2%, YOLOv11n by 2.4. Additionally, its computational complexity is minimized to 5.4 GFLOPs, representing reductions of approximately 5.3%, 6.7%, 53.1%, 30.5%, and 94.9% compared to YOLOv11n, YOLOv8n, YOLOv6n, YOLOv5n, and Rt-detr, respectively.

**Supplementary Figure S3** further illustrates the detailed training performance trends of each model. The precision, recall, mAP_50_, and mAP_95_ metrics for all models rapidly increase within the first 100 epochs and subsequently stabilize. Among them, the EDGE-MSE-YOLOv11 consistently maintains superior performance across all metrics, achieving higher stable values earlier and more steadily compared to other models. Specifically, the improvements in recall and mAP_95_ metrics are especially pronounced, clearly separating EDGE-MSE-YOLOv11 from other models after approximately 50 epochs. This indicates that the introduced enhancements significantly improve the model’s ability to accurately detect dense small targets from early training stages.

**Figure 11:**
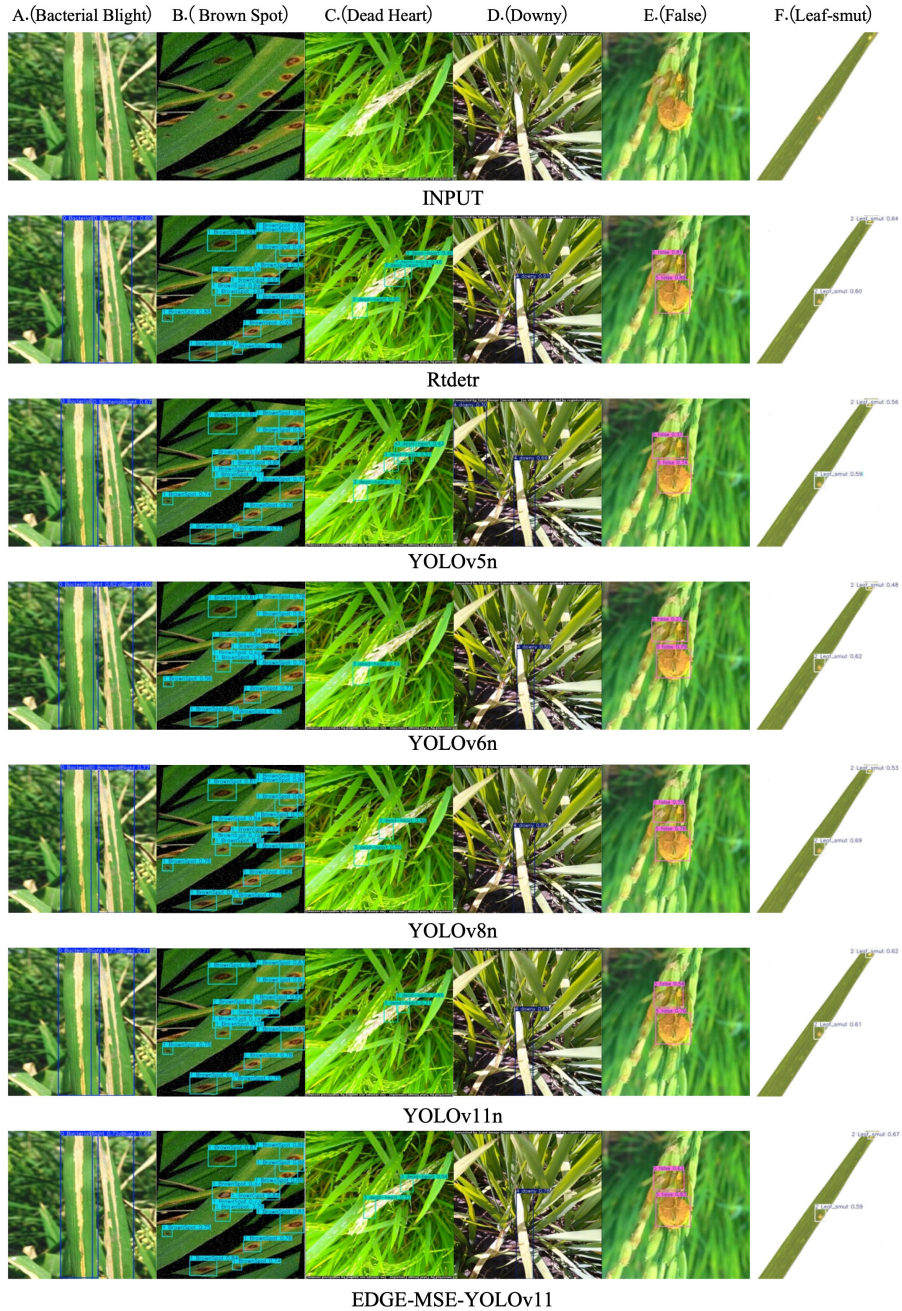
Detection effect of different models on the rice disease dataset: **(A)** Bacterial Blight, **(B)** Brown Spot, **(C)** Dead Heart, **(D)** Downy, **(E)** False, **(F)** Leaf-smut.

To qualitatively assess the detection performance of the rice disease recognition model EDGE-MSE-YOLOv11 proposed in this study, a comparative analysis was performed on the detection results of rice disease images from the test set using RT-DETR, YOLOv5n, YOLOv6n, YOLOv8n, YOLOv11n, and EDGE-MSE-YOLOv11. The detection results for various rice diseases, including A (Bacterial Blight), B (Brown Spot), C (Leaf Smut), D (Dead Heart), E (Downy), and F (False), are shown in **Figure 11**. From the detection results of RT-DETR, it is observed that the model performs well in detecting certain diseases but often struggles to identify small or localized lesion regions, with some prediction boxes showing imprecise positioning. Compared to RT-DETR, YOLOv5n and YOLOv6n show higher accuracy in detecting prominent lesions; however, they may still miss or misclassify disease types with significant variations in boundary shapes or appearances. YOLOv11n shows precise localization across various disease types, with prediction boxes closely matching diseased regions. This indicates that improvements in its network structure and feature fusion have enhanced disease detection. The enhanced EDGE-MSE-YOLOv11 model accurately marks diseased regions even when lesion edges are blurred, shapes are similar, or color differences are minimal. It shows superior ability in detecting small targets and complex backgrounds. These results indicate that, through the synergistic effects of multiple modules, the model effectively extracts key disease features while suppressing background interference, significantly reducing false and missed detection rates.

These results highlight that the enhanced EDGE-MSE-YOLOv11 model effectively extracts key disease features, suppresses background interference, and significantly reduces false and missed detection rates, all while maintaining low computational complexity and a compact size, making it well-suited for efficient deployment on resource-constrained edge devices, especially for detecting dense small targets.

### 4.4 Comparison with existing methods

To further demonstrate the effectiveness and advancement of the proposed EDGE-MSE-YOLOv11 model, we conducted a comparative analysis against several recently published rice disease detection models. As summarized in **Table 5**, the comparison includes key performance indicators such as mAP metrics, model size, parameter count, and computational complexity.

**Table 5:** Comparison with existing rice disease detection models.

Method	mAP50/%	mAP95/%	Model Size/M	Params/M	FLOPs/G
RDRM-YOLO (Li et al., 2025)	93.5	–	7.9	–	–
GDS-YOLO (Huang et al., 2025a)	85.3	–	–	8.97	7.3
YOLOv7-Tiny (Cheng et al., 2024)	92.2	–	–	12.2	–
**EDGE-MSE-YOLOv11 (Ours)**	**92.6**	**70.3**	**4.4**	**7.86**	**5.4**

The bold values represent the highest performance metrics achieved in each respective category.

For instance (Li et al., 2025), proposed RDRM-YOLO for rice disease detection under complex environmental conditions. Although their model achieved a high mAP50 of 93.5%, the model size reached 7.9 MB, which is notably larger than that of EDGE-MSE-YOLOv11. Similarly (Huang et al., 2025a), introduced GDS-YOLO by integrating GsConv, Dysample, SCAM, and WIoU v3, achieving an mAP50 of 85.3%, with a parameter count of 8.97M and computational complexity of 7.3 GFLOPs—both higher than our approach. In another study (Cheng et al., 2024), presented an enhanced YOLOv7-Tiny model, which reached an mAP50 of 92.2% with a large parameter size of 12.2M and a single-image inference time of 26.4 ms, satisfying real-time detection requirements but at the cost of model compactness.

In contrast, our proposed EDGE-MSE-YOLOv11 achieves a competitive mAP50 of 92.6% and a superior mAP95 of 70.3%, while maintaining a compact model size of only 4.4 MB, with 7.86M parameters and just 5.4 GFLOPs. These results highlight the effectiveness of the proposed Tri-Module Lightweight Perception Mechanism (TMLPM) and its strong balance between accuracy, model size, and efficiency. Such performance makes EDGE-MSE-YOLOv11 particularly suitable for real-time, resource-constrained applications in precision agriculture.

### 4.5 Design of a rice disease visualization system

**Figure 12:**
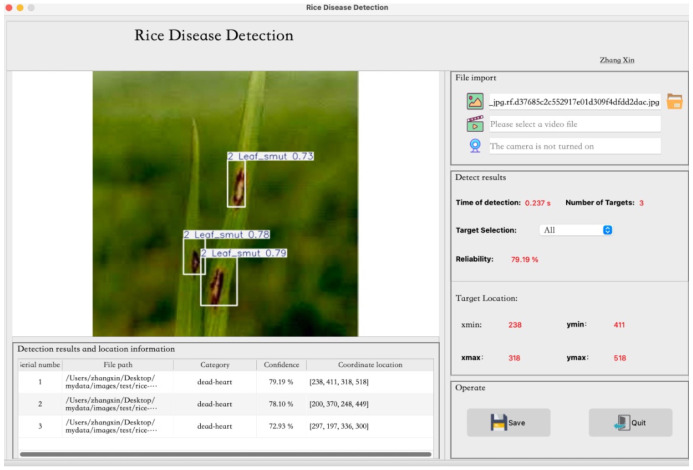
Comparison of improvement effects.

The trained improved model is converted into the appropriate file format and loaded using the PyTorch acceleration inference framework, then deployed on a Flask server. The user interface (UI) design, model invocation, and debugging are performed using the VSCode development tool to build and run the visual system. The development environment utilizes PyQt5 version 5.15.2, with Python 3.9.20 as the programming language. Users can upload images to select the ones they wish to detect, which are then transmitted to the Flask server for processing. The EDGE-MSE-YOLOv11 model is applied to capture and recognize rice disease images, with the recognition results returned in real time to the front-end interface (as shown in **Figure 12**). Additionally, the system supports video and camera detection features, displaying the location, quantity, reliability, and processing time of the detected targets, thus providing users with a comprehensive solution for disease identification.

## 5 Conclusion

This study proposes EDGE-MSE-YOLOv11, a novel lightweight rice disease detection model based on a unified Tri-Module Lightweight Perception Mechanism (TMLPM). This mechanism integrates three core components: multi-scale feature fusion (C3K2_MSEIE), attention-guided feature refinement (SimAM), and efficient spatial downsampling (ADown), which significantly enhance the model’s ability to detect multi-scale and small disease targets under complex field conditions. Experimental results show that compared to the baseline YOLOv11n model, EDGE-MSE-YOLOv11 achieves improvements of 3.6%, 3.8%, 2.4%, and 6.6% in precision (from 85.6% to 89.2%), recall (from 82.6% to 86.4%), mAP50 (from 90.2% to 92.6%), and mAP95 (from 63.7% to 70.3%), respectively. Meanwhile, the model’s parameter count is reduced from 8.55M to 7.86M and computational cost decreases from 5.7 GFLOPs to 5.4 GFLOPs, corresponding to reductions of approximately 0.69M parameters and 0.3 GFLOPs. In practical deployment scenarios, the model outperforms several state-of-the-art lightweight detectors including RT-DETR, YOLOv5n, YOLOv6n, YOLOv8n, and YOLOv11n, achieving a superior balance between high-speed inference at 111.6 FPS and excellent detection performance, thereby meeting the demands of intelligent agriculture applications.

The proposed model has been successfully deployed as a client-side application for real-time disease detection in agricultural environments, processing image data from cameras and other sensors with minimal computational overhead. In the future, as smart agriculture applications continue to evolve, we plan to extend the model to mobile platforms to provide on-site, smartphone-based disease detection for agricultural practitioners. Nonetheless, limitations remain. EDGE-MSE-YOLOv11 may face challenges in extremely complex field environments, such as ultra-small lesions, occluded leaves, or overlapping disease symptoms, which could affect detection reliability. Moreover, the model is currently evaluated on a single-domain dataset, and its cross-domain generalization to other crops or geographic regions requires further validation.

To overcome these limitations, future work will focus on the following aspects: (1) Enhancing deployment efficiency and hardware adaptability by leveraging techniques such as LoRA-based fine-tuning, quantization-aware training (QAT), and structured pruning; (2)) Improving spatial reasoning and disease pattern recognition by incorporating MobileViT or graph neural network (GNN)-based modules into the TMLPM framework; (3) Expanding training datasets to cover a wider range of crops, environments, and disease types, thereby building a robust and generalizable multi-crop, multi-disease detection system specifically designed for precision agriculture applications.

## Data Availability

The raw data supporting the conclusions of this article will be made available by the authors, without undue reservation.

## References

[B1] Alkhammash E. H. (2025). A comparative analysis of yolov9, yolov10, yolov11 for smoke and fire detection. Fire 8(1), 2571–6255. doi: 10.3390/fire8010026

[B2] Bist R. B. Subedi S. Yang X. Chai L. (2023). A novel yolov6 object detector for monitoring piling behavior of cage-free laying hens. AgriEngineering 5, 905–923. doi: 10.3390/agriengineering5020056

[B3] Chattopadhay A. Sarkar A. Howlader P. Balasubramanian V. N. (2018). “Grad-cam++: Generalized gradient-based visual explanations for deep convolutional networks,” in 2018 IEEE winter conference on applications of computer vision (WACV). 839–847 (IEEE).

[B4] Chen Z. Cai Y. Liu Y. Liang Z. Chen H. Ma R.. (2025). Towards end-to-end rice row detection in paddy fields exploiting two-pathway instance segmentation. Comput. Electron. Agric. 231, 109963. doi: 10.1016/j.compag.2025.109963

[B5] Cheng D. Zhao Z. Feng J. (2024). Rice diseases identification method based on improved yolov7-tiny. Agriculture 14, 709. doi: 10.3390/agriculture14050709

[B6] Deng J. Liu S. Chen H. Chang Y. Yu Y. Ma W.. (2025). A precise method for identifying 3d circles in freeform surface point clouds. IEEE Trans. Instrumentation Measurement. 74, 1–13. doi: 10.1109/TIM.2025.3547492

[B7] Fang S. Chen C. Li Z. Zhou M. Wei R. (2024). Yolo-adual: A lightweight traffic sign detection model for a mobile driving system. World Electric Vehicle J. 15, 323. doi: 10.3390/wevj15070323

[B8] Fu H. Song G. Wang Y. (2021). Improved yolov4 marine target detection combined with cbam. Symmetry 13, 623. doi: 10.3390/sym13040623

[B9] Guan S. Lin Y. Lin G. Su P. Huang S. Meng X.. (2024). Real-time detection and counting of wheat spikes based on improved yolov10. Agronomy 14, 1936. doi: 10.3390/agronomy14091936

[B10] He Y. Yu H. Liu X. Yang Z. Sun W. Anwar S.. (2021). Deep learning based 3d segmentation: A survey.

[B11] Huang Y. Feng X. Han T. Song H. Liu Y. Bao M. (2025a). Gds-yolo: A rice diseases identification model with enhanced feature extraction capability. IET Image Process. 19, e70034. doi: 10.1049/ipr2.70034

[B12] Huang Y. Liu Z. Zhao H. Tang C. Liu B. Li Z.. (2025b). Yolo-ysts: An improved yolov10nbased method for real-time field pest detection. Agronomy 15, 575. doi: 10.3390/agronomy15030575

[B13] Jegham N. Koh C. Y. Abdelatti M. Hendawi A. (2024). Evaluating the evolution of yolo (you only look once) models: A comprehensive benchmark study of yolo11 and its predecessors.

[B14] Jiang D. Wang H. Li T. Gouda M. A. Zhou B. (2025). Real-time tracker of chicken for poultry based on attention mechanism-enhanced yolo-chicken algorithm. Comput. Electron. Agric. 237, 110640. doi: 10.1016/j.compag.2025.110640

[B15] Jocher G. Stoken A. Chaurasia A. Borovec J. Kwon Y. Michael K.. (2021). ultralytics/yolov5: v6. 0-yolov5n’nano’models, roboflow integration, tensorflow export, opencv dnn support (Zenodo: Zenodo).

[B16] Khanam R. Hussain M. (2024). Yolov11: An overview of the key architectural enhancements.

[B17] Kutyrev A. Khort D. Smirnov I. Zubina V. (2025). “Uav-based sustainable orchard management: Deep learning for apple detection and yield estimation,” in E3S Web of Conferences (EDP Sciences), Vol. 614. 03021.

[B18] Lee Y.-S. Patil M. P. Kim J. G. Seo Y. B. Ahn D.-H. Kim G.-D. (2025). Hyperparameter optimization for tomato leaf disease recognition based on yolov11m. Plants 14, 653. doi: 10.3390/plants14050653, PMID: 40094534 PMC11901684

[B19] Li R. Li Y. Qin W. Abbas A. Li S. Ji R.. (2024). Lightweight network for corn leaf disease identification based on improved yolo v8s. Agriculture 14, 220. doi: 10.3390/agriculture14020220

[B20] Li P. Zhou J. Sun H. Zeng J. (2025). Rdrm-yolo: A high-accuracy and lightweight rice disease detection model for complex field environments based on improved yolov5. Agriculture 15, 479. doi: 10.3390/agriculture15050479

[B21] Liu J.-J. Hou Q. Liu Z.-A. Cheng M.-M. (2022). Poolnet+: Exploring the potential of pooling for salient object detection. IEEE Trans. Pattern Anal. Mach. Intell. 45, 887–904. doi: 10.1109/TPAMI.2021.3140168, PMID: 34982676

[B22] Liu Q. Huang W. Duan X. Wei J. Hu T. Yu J.. (2023). Dsw-yolov8n: A new underwater target detection algorithm based on improved yolov8n. Electronics 12, 3892. doi: 10.3390/electronics12183892

[B23] Lucas J. (2011). Advances in plant disease and pest management. J. Agric. Sci. 149, 91–114. doi: 10.1017/S0021859610000997

[B24] Martinelli F. Scalenghe R. Davino S. Panno S. Scuderi G. Ruisi P.. (2015). Advanced methods of plant disease detection. a review. Agron. Sustain. Dev. 35, 1–25. doi: 10.1007/s13593-014-0246-1

[B25] Mathew M. P. Mahesh T. Y. (2022). Leaf-based disease detection in bell pepper plant using yolo v5. Signal Image Video Process. 16(3), 1–7. doi: 10.1007/s11760-021-02024-y

[B26] Mew T. W. Leung H. Savary S. Vera Cruz C. M. Leach J. E. (2004). Looking ahead in rice disease research and management. Crit. Rev. Plant Sci. 23, 103–127. doi: 10.1080/07352680490433231

[B27] Rao H. Zhan H. Wang R. Yu J. (2025). A lightweight and enhanced yolo11-based method for small object surface defect detection. doi: 10.21203/rs.3.rs-6093937/v1

[B28] Sapkota R. Karkee M. (2024a). Integrating yolo11 and convolution block attention module for multi-season segmentation of tree trunks and branches in commercial apple orchards.

[B29] Sapkota R. Karkee M. (2024b). Yolo11 and vision transformers based 3d pose estimation of immature green fruits in commercial apple orchards for robotic thinning. doi: 10.36227/techrxiv.173014437.72236643/v1

[B30] Sapkota R. Meng Z. Churuvija M. Du X. Ma Z. Karkee M. (2024). Comprehensive performance evaluation of yolo11, yolov10, yolov9 and yolov8 on detecting and counting fruitlet in complex orchard environments. doi: 10.36227/techrxiv.172954111.18265256/v1

[B31] Savary S. Ficke A. Aubertot J.-N. Hollier C. (2012). Crop losses due to diseases and their implications for global food production losses and food security. Food Secur. 4, 519–537. doi: 10.1007/s12571-012-0200-5

[B32] Shoaib M. Shah B. Ei-Sappagh S. Ali A. Ullah A. Alenezi F.. (2023). An advanced deep learning models-based plant disease detection: A review of recent research. Front. Plant Sci. 14, 1158933. doi: 10.3389/fpls.2023.1158933, PMID: 37025141 PMC10070872

[B33] Sirisha U. Praveen S. P. Srinivasu P. N. Barsocchi P. Bhoi A. K. (2023). Statistical analysis of design aspects of various yolo-based deep learning models for object detection. Int. J. Comput. Intell. Syst. 16, 126. doi: 10.1007/s44196-023-00302-w

[B34] Soeb M. J. A. Jubayer M. F. Tarin T. A. Al Mamun M. R. Ruhad F. M. Parven A.. (2023). Tea leaf disease detection and identification based on yolov7 (yolo-t). Sci. Rep. 13, 6078. doi: 10.1038/s41598-023-33270-4, PMID: 37055480 PMC10102080

[B35] Song H. Yan Y. Deng S. Jian C. Xiong J. (2024). Innovative lightweight deep learning architecture for enhanced rice pest identification. Physica Scripta 99, 096007. doi: 10.1088/1402-4896/ad69d5

[B36] Suzuki K. Horiba I. Sugie N. (2003). Neural edge enhancer for supervised edge enhancement from noisy images. IEEE Trans. Pattern Anal. Mach. Intell. 25, 1582–1596. doi: 10.1109/TPAMI.2003.1251151

[B37] Tank K. H. Ghanem M. C. Vassilev V. Ouazzane K. (2025). Synchronization, optimization, and adaptation of machine learning techniques for computer vision in cyber-physical systems: a comprehensive analysis 1–31. doi: 10.20944/preprints202501.0521.v1

[B38] Wang C. Han J. Liu C. Zhang J. Qi Y. (2025b). Lehp-detr: A model with backbone improved and hybrid encoding innovated for flax capsule detection. iScience 28, 2589–0042. doi: 10.1016/j.isci.2024.111558, PMID: 39877068 PMC11773470

[B39] Wang S. Jiang H. Yang J. Ma X. Chen J. Li Z.. (2024). Lightweight tomato ripeness detection algorithm based on the improved rt-detr. Front. Plant Sci. 15, 1415297. doi: 10.3389/fpls.2024.1415297, PMID: 39036358 PMC11257922

[B40] Wang J. Ma S. Wang Z. Ma X. Yang C. Chen G.. (2025c). Improved lightweight yolov8 model for rice disease detection in multi-scale scenarios. Agronomy 15, 445. doi: 10.3390/agronomy15020445

[B41] Wang S. Xu D. Liang H. Bai Y. Li X. Zhou J.. (2025d). Advances in deep learning applications for plant disease and pest detection: A review. Remote Sens. 17, 698. doi: 10.3390/rs17040698

[B42] Wang B. Yang M. Cao P. Liu Y. (2025a). A novel embedded cross framework for high-resolution salient object detection. Appl. Intell. 55, 277. doi: 10.1007/s10489-024-06073-x

[B43] Wu T. Dong Y. (2023). Yolo-se: Improved yolov8 for remote sensing object detection and recognition. Appl. Sci. 13, 12977. doi: 10.3390/app132412977

[B44] Xiang Z. Wan X. Xu L. Yu X. Mao Y. (2024). A training-free latent diffusion style transfer method. Information 15, 588. doi: 10.3390/info15100588

[B45] Xue Z. Xu R. Bai D. Lin H. (2023). Yolo-tea: A tea disease detection model improved by yolov5. Forests 14, 415. doi: 10.3390/f14020415

[B46] Yan J. Zeng Y. Lin J. Pei Z. Fan J. Fang C.. (2024). Enhanced object detection in pediatric bronchoscopy images using yolo-based algorithms with cbam attention mechanism. Heliyon 10, 839–847. doi: 10.1016/j.heliyon.2024.e32678, PMID: 39021922 PMC11252869

[B47] Yang L. Guo F. Zhang H. Cao Y. Feng S. (2024). Research on lightweight rice false smut disease identification method based on improved yolov8n model. Agronomy 14, 1934. doi: 10.3390/agronomy14091934

[B48] Ye R. Shao G. He Y. Gao Q. Li T. (2024). Yolov8-rmda: Lightweight yolov8 network for early detection of small target diseases in tea. Sensors 24, 2896. doi: 10.3390/s24092896, PMID: 38733002 PMC11086262

[B49] Zhai X. Huang Z. Li T. Liu H. Wang S. (2023). Yolo-drone: an optimized yolov8 network for tiny uav object detection. Electronics 12, 3664. doi: 10.3390/electronics12173664

[B50] Zhang M. Ye S. Zhao S. Wang W. Xie C. (2025). Pear object detection in complex orchard environment based on improved yolo11. Symmetry 17, 255. doi: 10.3390/sym17020255

[B51] Zhao B. Chang L. Liu Z. (2025). Fast-yolo network model for x-ray image detection of pneumonia. Electronics 14, 903. doi: 10.3390/electronics14050903

[B52] Zhao Y. Lv W. Xu S. Wei J. Wang G. Dang Q.. (2024). “Detrs beat yolos on real-time object detection,” in Proceedings of the IEEE/CVF conference on computer vision and pattern recognition. 16965–16974.

[B53] Zhou L. Cai J. Ding S. (2023). The identification of ice floes and calculation of sea ice concentration based on a deep learning method. Remote Sens. 15, 2663. doi: 10.3390/rs15102663

