# Supplementary material for: TriPerceptNet: a lightweight multi-scale enhanced YOLOv11 model for accurate rice disease detection in complex field environments
Source: Front Plant Sci. 2025 Sep 4;16:1614929. doi: 10.3389/fpls.2025.1614929 (PMC12445169; doi:10.3389/fpls.2025.1614929)
Supplement: Supplementary file 1 [file DataSheet1.pdf]

Supplementary Material

0.1 Figures

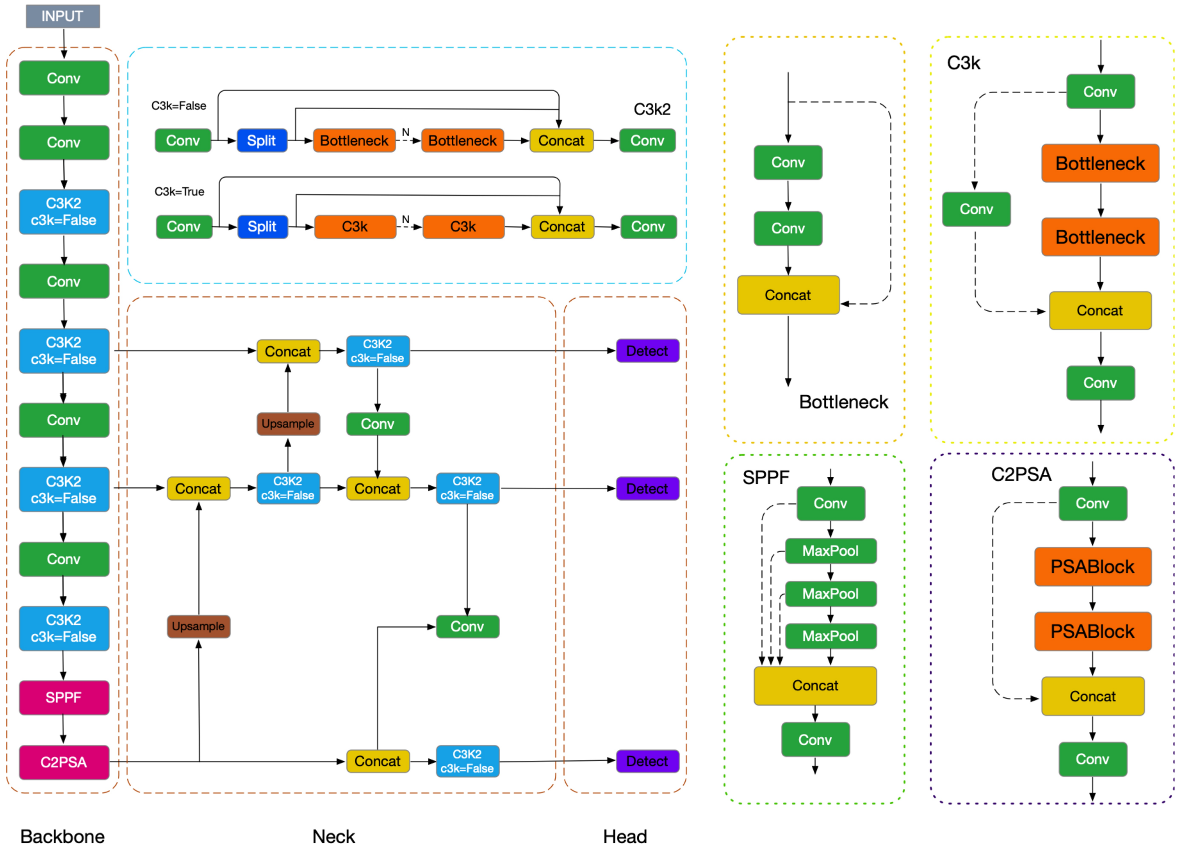

Figure S1. YOLOv11 Structure Diagram

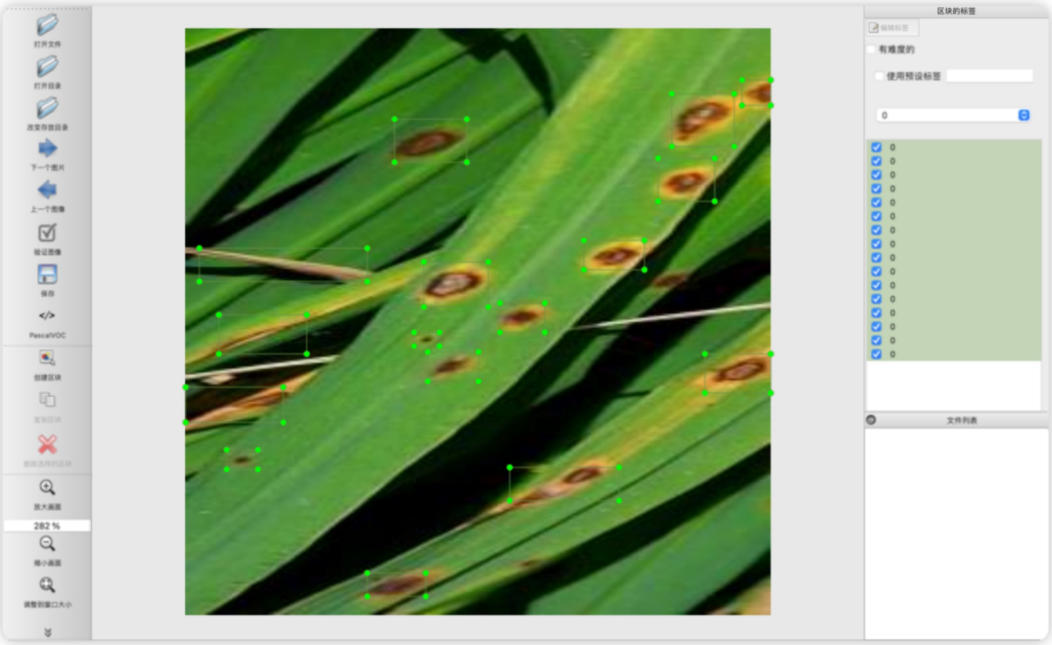

Figure S2. Diagram of the Annotation Process

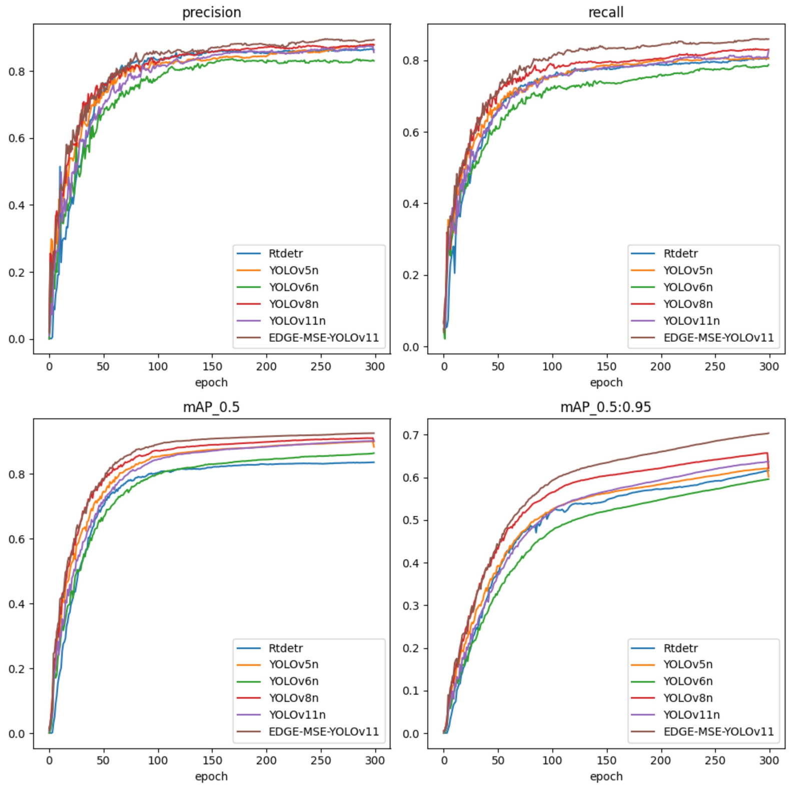

Figure S3. Comparison of Improvement Effects
